# Effects of anthocyanin supplementation in diet on glycemic and related cardiovascular biomarkers in patients with type 2 diabetes: a systematic review and meta-analysis of randomized controlled trials

**DOI:** 10.3389/fnut.2023.1199815

**Published:** 2023-09-22

**Authors:** Ting Mao, F. N. U. Akshit, Maneesha S. Mohan

**Affiliations:** Alfred Dairy Science Laboratory, Department of Dairy and Food Science, South Dakota State University, Brookings, SD, United States

**Keywords:** anthocyanins, polyphenol, glycemic control, insulin resistance, lipid profile, type 2 diabetes, blood pressure

## Abstract

**Purpose:**

This study is the first systematic review and meta-analysis based on RCTs on the effects of anthocyanins on patients with type 2 diabetes mellitus (T2DM) and the effect on T2DM-related cardiovascular disease.

**Methods:**

RCTs published in English from five electronic databases were evaluated for glycated hemoglobin (HbA_1c_), fasting blood glucose (FBG), 2-h postprandial blood glucose, fasting insulin, model assessment for insulin resistance, triglycerides (TG), total cholesterol (TC), high-density lipoprotein (HDL) cholesterol, low-density lipoprotein (LDL) cholesterol, systolic blood pressure, and diastolic blood pressure. The quality of the studies was rated (Cochrane Risk of Bias tool) and weighted mean differences were calculated (DerSimonian-Laird model with random effects). Leave-one-out sensitivity, subgroup, and publication bias analyses were conducted. The strength of the evidence was rated according to the GRADE guidelines.

**Results:**

In all, 13 RCTs were analyzed out of the 239 identified studies, with a duration longer than 4 weeks (703 participants with T2DM). Our findings indicate that a median dose of 320 mg/day anthocyanins, either from fruit extracts or pure supplements, for a median intervention length of 8 weeks significantly reduced HbA_1c_ [Weighted Mean Difference (WMD) −0.31, *p* = 0.00], FBG (WMD −0.63, *p* = 0.00), 2-h postprandial glucose (WMD −1.60, *p* = 0.00), TG (WMD −0.45, *p* = 0.01), and LDL (WMD −0.26 *p* = 0.02). However, the effects of anthocyanins on fasting insulin, HOMA-IR, TC, HDL cholesterol, systolic blood pressure, and diastolic blood pressure in patients with T2DM were not statistically significant. Anthocyanins from fruit extracts or powder exhibited a higher reduction of HbA_1c_ compared to pure anthocyanin supplements.

**Conclusion:**

The significant improvements in glycemic parameters and lipid profile, suggest the benefits of anthocyanins, especially from fruit extract or powder, in the management of T2DM, and their ability to delay the onset of lipid disorder-related diseases such as cardiovascular disease associated with T2DM. The mechanism behind this reduction in glycemic markers could be attributed to the antioxidant and anti-inflammatory activity of anthocyanins. Further research with well-designed RCTs is required to determine the optimal dosage of anthocyanins for the treatment of T2DM and to comprehend the consequences.

## 1. Introduction

Type 2 diabetes mellitus (T2DM) accounts for approximately 90% of all occurrences of diabetes in the world and is characterized by insulin resistance and hyperglycemia (1–3). T2DM is a rising public health concern globally and the number of individuals with T2DM is expected to surge to 592 million by 2035, from only 108 million in 1980 (4, 5). An estimated 1.5 million reported deaths per year have been associated with T2DM globally (6). Unhealthy lifestyle, dietary patterns, sedentarism, alcohol consumption, and smoking, have been related to fat accumulation in tissues including in the pancreas, allied with the development of insulin resistance or impaired insulin secretion by the pancreatic beta cells, preceding T2DM diagnosis by up to 15 years (2, 7, 8). The incidence of T2DM has been found to increase with age, with the highest incidences in ages above 45 years. However, recently T2DM has been increasingly seen in young adults due to changes in lifestyle and a sedentary lifestyle (2).

**Figure 1:**
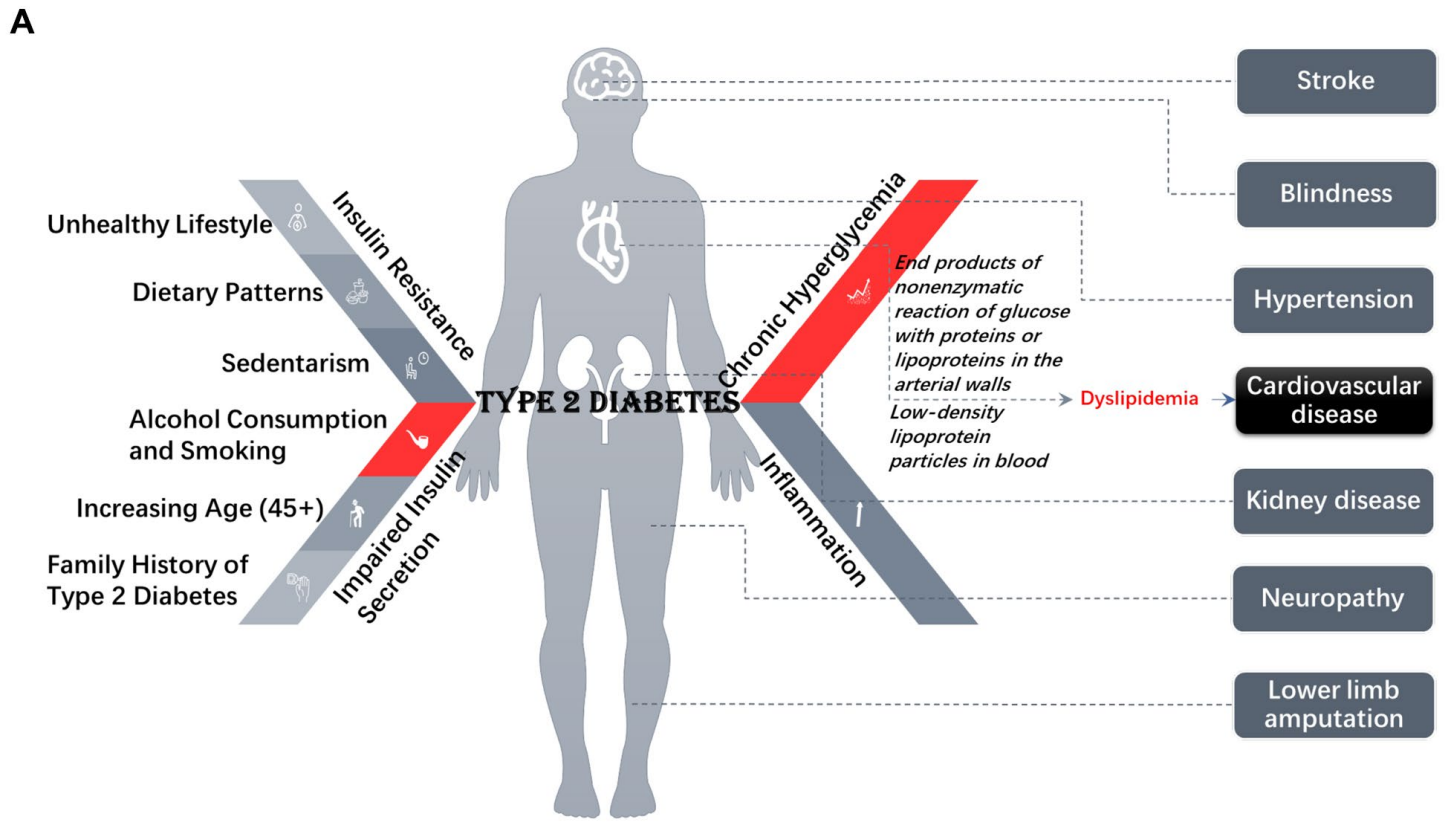

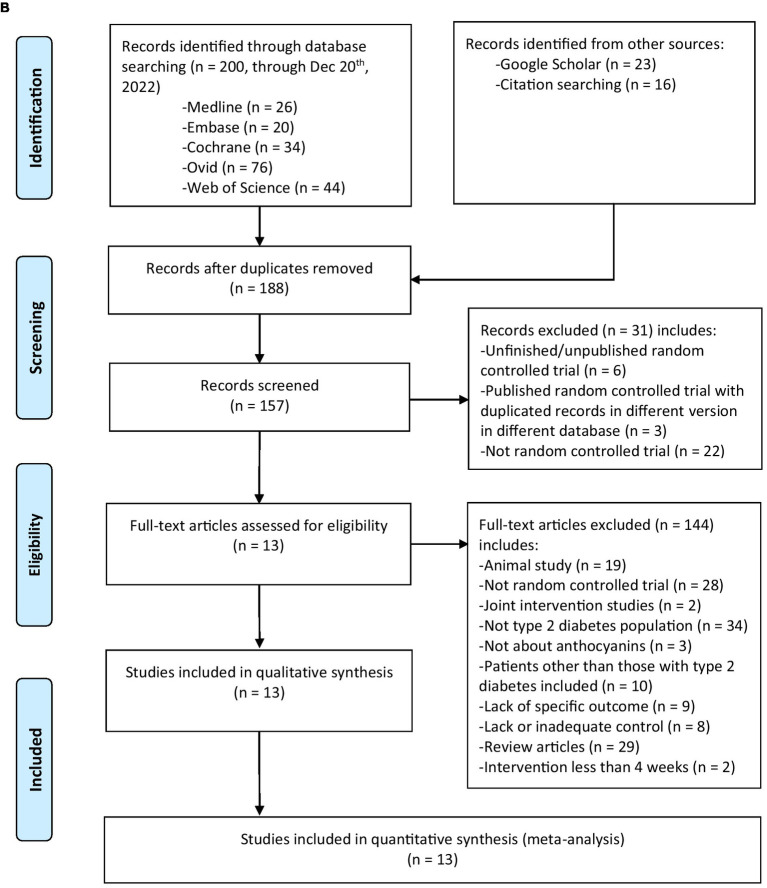

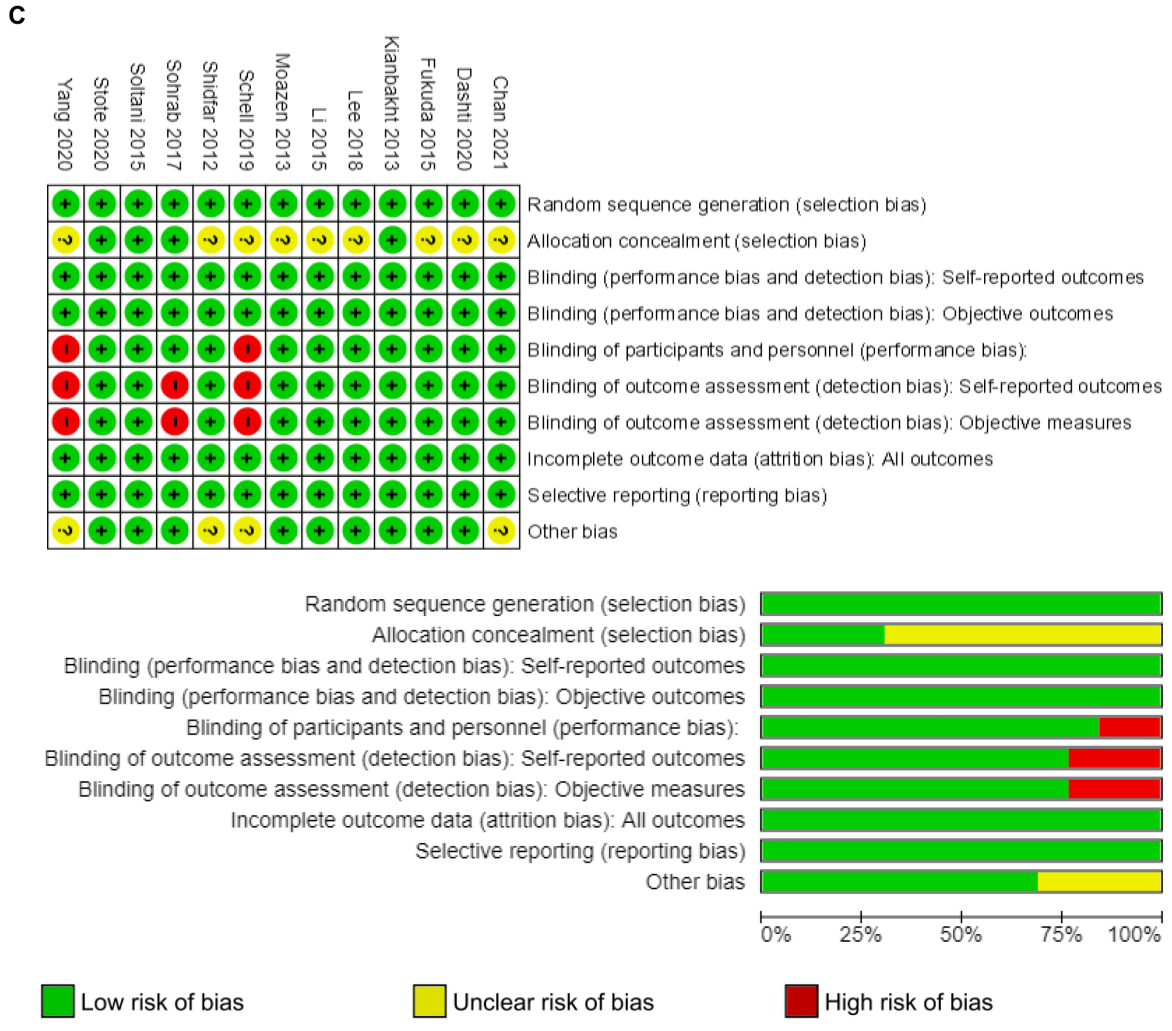
**(A)** The causes and consequences related to type 2 diabetes. **(B)** Flowchart of the search results [based on PRISMA 2009 flow diagram guidelines (45)]. **(C)** Literature quality evaluation chart based on individual Cochrane risk of bias assessment.

Long-term hyperglycemia leads to the formation of advanced glycosylation end products of the non-enzymatic reaction of glucose with proteins or lipoproteins in the arterial walls and low-density lipoprotein particles in the blood, resulting in dyslipidemia, eventually leading to cardiovascular disorder, as well as nephropathy, retinopathy, and neuropathy (9, 10). Many inflammation markers including cytokines, interleukins, chemokines, and adipokines, serve as the pathologic links with different mechanisms associated with T2DM and also contribute to imbalances in lipid metabolism (11), dysbiosis (12, 13), and atherosclerosis (14). Thus, T2DM patients with impaired metabolic homeostasis are more likely to experience complications including cardiovascular disease, stroke, hypertension, kidney failure, blindness, lower limb amputation, and premature death (3, 9, 15–17), as represented in Figure 1A. Various medications are available to manage glycemic parameters, however, their impact on long term effect, well-being, and safety of patients with T2DM is debatable (9).

In comparison to pharmaceutical therapies, dietary changes, and supplements have been suggested as sustainable, safe, and cost-effective ways to manage T2DM. Although the most widely recommended approach is the supplementation of diet with fiber, there is growing evidence indicating that polyphenols impact blood glucose to varying degrees, depending on the type of polyphenols, and help regulate and avoid diabetes complications (6, 18, 19). Among them, anthocyanins, which belong to the flavonoid class of polyphenols, are widely present in seeds, flowers, fruits, and leaves of a large variety of plants (20). They give flowers and fruits their pink, red, blue, or purple color and are present in the vacuolar sap of epidermal tissues (21). The human diet contains six different forms of anthocyanins: cyanidin, delphinidin, malvidin, pelargonidin, peonidin, and petunidin. Black Chokeberry (400–1,500 mg/100 g fresh fruit), blueberries (60–300 mg/100 g fresh fruit), blackcurrant (100–500 mg/100 g fresh fruit), blackberries (50–350 mg/100 g fresh fruit), and purple corn (≥1,500 mg/100 g fresh fruit) are some examples of fruits with varying anthocyanin contents (22). The production of these fruits and their polyphenol content varies with the geographical location, and this also affects the intake of anthocyanins by the population in that specific location, in addition to the *per capita* consumption. Huge variations in the average daily intake of polyphenols have been documented in different geographical regions, with 10.3 mg in the USA, 44.1–64.9 mg in Italy, and 47 mg in Finland. However, the increased consumption of berries in the latter two countries is the reason for higher daily intake values (4, 23).

Recently, the benefits of anthocyanins in preventing or treating T2DM and cardiovascular disease have been studied extensively (2, 5, 22, 24–28). These investigations revealed the potential of anthocyanins in improving glycemic management and lipid profile in healthy adults as well as in a limited subset of patients suffering from conditions such as obesity or being overweight, hyperlipidemia, hypercholesterolemia, metabolic syndrome, and T2DM. However, there are no systematic reviews or meta-analyses based on randomized controlled trials (RCTs) evaluating the benefits of anthocyanins on glycemic control, insulin resistance, and associated cardiovascular parameters (lipid profile and blood pressure) in individuals with T2DM. The mechanism of anthocyanins in decreasing the onset and development of T2DM is linked to the antioxidant and anti-inflammatory properties of anthocyanins, mainly manifested in the ability to scavenge free radicals, induce secretion of glucagon-like peptide I (GLP-I), inhibit xanthine oxidase, chelate iron or copper, and target arachidonic acid, nuclear factor kappa B (NF-κB), and tumor necrosis factor-alpha (TNF-α) (5, 29–32). Based on these findings, researchers further associated the consumption of anthocyanins with the lowering of low-density lipoprotein (LDL) cholesterol, low-grade systemic inflammation, and reactive oxygen species-related damage (18, 25, 33–35). These findings suggest that anthocyanins have the potential to lower the risk of cardiovascular disease resulting from or associated with T2DM, in addition to the effects on T2DM. Cardiovascular disease is the most common comorbidity and the primary cause of mortality in patients with T2DM, as it is prevalent in T2DM patients 2- to 4-fold more than in the general population (36–40). Globally, approximately 32.2% of patients with T2DM have some form of cardiovascular disease (41). Hence, more studies are required to further understand the impact of anthocyanins on glycemic and cardiovascular indicators in patients with T2DM.

In this review, therefore, we conduct a thorough meta-analysis of RCTs to examine the effects of anthocyanins (either from fruit extracts or pure anthocyanins supplements) on glycemic control (including FBG, HbA1c, insulin resistance, and 2-h postprandial glucose) along with investigating the effects on biomarkers related to cardiovascular health indicators affected by T2DM (including lipid profile and blood pressure).

## 2. Methods

### 2.1. Search strategy and selection criteria

The review was registered with PROSPERO, CRD42023387912, following the Cochrane Handbook for Systematic Reviews of Interventions guidelines (42) and Preferred Reporting Items for Systematic Reviews and Meta-Analyses (PRISMA) reporting requirements (43).

We selected authentic RCTs that investigated the impact of anthocyanins on glycemic control and lipid profile in patients with T2DM, from five electronic databases, namely, PubMed (Medline), Embase, Cochrane library, Ovid, and Web of Science. Studies published in English before 20 August 2023 were taken into consideration. The following combined text and Medical subject headings (MeSH) terms were used: “anthocyanins” and “diabetes mellitus, type 2.” The search approach used for PubMed was (“Anthocyanins”[Mesh] OR Anthocyanin OR Leucoanthocyanidins OR Anthocyanidins OR Anthocyanidin) AND (“Diabetes Mellitus, Type 2”[Mesh] OR Diabetes Mellitus, Noninsulin-Dependent OR Diabetes Mellitus, Ketosis-Resistant OR Diabetes Mellitus, Ketosis Resistant OR Ketosis-Resistant Diabetes Mellitus OR Diabetes Mellitus, Noninsulin Dependent OR Diabetes Mellitus, Noninsulin-Dependent OR Non-Insulin-Dependent Diabetes Mellitus OR Diabetes Mellitus, Stable OR Stable Diabetes Mellitus OR Diabetes Mellitus, Type II OR NIDDM OR Diabetes Mellitus, Noninsulin Dependent OR Diabetes Mellitus, Maturity-Onset OR Diabetes Mellitus, Maturity Onset OR Maturity-Onset Diabetes Mellitus OR Maturity Onset Diabetes Mellitus OR MODY OR Diabetes Mellitus, Slow-Onset OR Diabetes Mellitus, Slow Onset OR Slow-Onset Diabetes Mellitus OR Type 2 Diabetes Mellitus OR Noninsulin-Dependent Diabetes Mellitus OR Noninsulin Dependent Diabetes Mellitus OR Maturity-Onset Diabetes OR Diabetes, Maturity-Onset OR Maturity Onset Diabetes OR Type 2 Diabetes OR Diabetes, Type 2 OR Diabetes Mellitus, Adult-Onset OR Adult-Onset Diabetes Mellitus OR Diabetes Mellitus, Adult Onset) AND (randomized controlled trial [Publication Type] OR randomized [Title/Abstract] OR placebo [Title/Abstract]). The search method was applied to all databases with no restriction on publication year. In order to find potentially qualified RCTs, we also looked through reference lists in earlier review articles and searched Google Scholar.

The PICOS (participants, intervention, comparator, outcomes, and study design) was used to determine the inclusion and exclusion criteria for eligible RCTs (Table 1).

**Table 1:** PICOS criteria for study eligibility.

PICOS	Inclusion	Exclusion
Participants	Participants were people with T2DM, regardless of their sex, age, race, nationality, number of years since the onset of T2DM, and body mass index (BMI)	Participants without T2DM, such as healthy population, and patients with hypertension or cardiovascular diseases; participants with type 2 diabetes complications; not specifying whether the subjects were receiving medications or undergoing other treatment.
Intervention	Intervention using dietary or supplemental anthocyanins.	Intervention without anthocyanins; study with joint intervention; intervention lacking adequate control; studies with less than a 4-week intervention period.
Comparator	Placebo	Intervention lacking adequate control.
Outcomes	At least one of the glycemic parameters, insulin resistance markers, and lipid profile, including FBG, 2-h postprandial glucose, HbA_1c_, fasting insulin, HOMA-IR, TG, TC, HDL cholesterol, and LDL cholesterol were investigated in the trial.	Studies lacking a specific outcome.
Study design	RCTs	Not RCTs; animal studies.

### 2.2. Data extraction

EndNote 20 was used to manage the identified studies. Following the removal of duplication, the titles and abstracts of all studies were reviewed by two separate researchers (TM and FA). RCTs that matched all the requirements for inclusion underwent a thorough examination. RCTs chosen for in-depth analysis obtained approval from both researchers.

For each included RCT, the following information was extracted: publication author, publication year, research geographical location, intervention/control participants number, age, sex, body mass index (BMI), duration of diabetes, study design (crossover or parallel), whether anti-diabetic or lipid-lowering medications or therapies were used during the intervention, source of anthocyanins, intervention dosage, intervention length, measured outcomes, and baseline and endpoint of each outcome including FBG, HbA_1c_, 2-h postprandial blood glucose, fasting insulin, HOMA-IR, triglycerides (TG), total cholesterol (TC), high-density lipoprotein (HDL) cholesterol, and LDL cholesterol, systolic blood pressure (SBP), and diastolic blood pressure (DBP). All the outcomes measured were continuous results and were extracted as mean ± standard deviation (SD).

### 2.3. Quality assessment of included RCTs

We used the Cochrane Risk of Bias Tool, to analyze and appraise the quality of each included RCT (44). For each trial that was included, six different categories of bias were assessed, including selection (random sequence generation and allocation concealment), performance, detection, attrition, reporting, and other biases. All of these biases were categorized as either having a low, high, or unclear risk. RCTs including more than half of biases with high risk were excluded in the following analysis.

### 2.4. Data analysis

To quantify the effect of anthocyanins on glycemic management, insulin resistance, lipid profile, and blood pressure in patients with T2DM, we evaluated the primary outcome HbA_1c_ and 10 secondary outcomes, namely, FBG, 2-h postprandial blood glucose, fasting insulin, HOMA-IR, TG, TC, HDL cholesterol, LDL cholesterol, SBP, and DBP. All these continuous parameters were analyzed by Stata (version 17). The mean difference before and after intervention in every outcome was conducted as a pooled analysis following a random-effects DerSimonian-Laird model to account for the increased uncertainty brought on by between-study variability.

Between-study heterogeneity was measured by the Cochran Q statistic and characterized using the I2 value. Values larger than 50% were regarded as significant between-study heterogeneity (45). If significant between-study heterogeneity was detected, the Galbraith plot and leave-one-out sensitivity were used to investigate the origins of heterogeneity. After the studies were removed for heterogeneity in each specific outcome, if there were more than 10 studies for the quantitative analysis, subgroup analysis would also be applied to figure out the sources of heterogeneity, and it was optional for the outcome including, less than 10 studies.

Funnel plots and Egger’s test to check publication bias were included in the analysis when there were more than 10 RCTs. Only Egger’s test was employed to analyze for publication bias in cases when fewer than 10 RCTs were included. In these studies, if the value of p from Egger’s test was less than 0.05, it was declared as proof of small study effects including between-study and publication biases. If publication bias was identified, a subsequent trim-and-fill procedure was utilized to fill the data of missing studies and compensate for asymmetry (46).

### 2.5. Evidence grading

To rate the degree of certainty of our findings, GRADE criteria were applied (47). If anthocyanins can significantly affect the selected outcomes, then we graded the evidence as either high, moderate, low, or very low according to the certainty assessment of study design, the risk of bias, inconsistency, indirectness, imprecision, and other considerations including publication bias, large effect, plausible confounding, and dose–response gradient. If the effects were not significant, the evidence was not graded.

## 3. Results

### 3.1. Search results

The search located 200 publications from PubMed (Medline), Embase, Web of Science, Cochrane Library, and Ovid. The flowchart for searching for the RCTs fulfilling the appropriate conditions for this study is shown in Figure 1B. An additional 39 papers were found by checking references from review papers identified during screening. Among them, 157 articles were reviewed in full and sought for retrieval. A total of 13 RCTs involving 703 participants with T2DM were eligible for the final analysis (9, 48–60). All of these articles investigated FBG, 9 of them evaluated HbA_1c_, involving 500 participants with T2DM (9, 48–50, 53–56, 59), 5 of them analyzed 2-h postprandial glucose levels in 309 participants with T2DM (9, 49, 51, 52, 54), 6 of them assessed fasting insulin in 315 participants with T2DM (50, 51, 53–56), and 4 of them explored HOMA-IR in 238 participants with T2DM (50, 54–56). Out of these articles, 8 studies analyzed the lipid profile, including TG (450 patients with T2DM) (9, 48, 50, 52–56), TC, HDL, and LDL (415 patients with T2DM) (9, 48, 50, 51, 53–56, 58). Out of these articles, 6 reported SBP and DBP in 265 patients with T2GM (9, 48, 50, 51, 53, 56).

### 3.2. Characteristics of selected RCTs

The different features of the selected RCTs are listed in Table 2, of which 2 were crossover RCTs (48, 51) and 11 were parallel RCTs (9, 49, 50, 52–57, 59, 60). These RCTs were carried out in an outpatient context, with 6 conducted in the Middle East, 5 in Asia, and 2 in North America. In the 13 RCTs that were included, a total of 703 patients with T2DM were investigated. They were 57 years old on average (range 37–73 years), with a mean BMI of 28.15 Kg/m2 (range 20.5–37.3), a mean baseline FBG level of 8.68 mmol/L (range 4.9–16.2), and mean baseline HbA_1c_ of 7.21% (range 4.3–9.51; Table 3). The median daily dose of anthocyanins supplements for all included RCTs was 320 mg (range 0.672–1,400) and the median length of intervention was 8 weeks (range 4–24 weeks).

**Table 2:** Features of the included RCTs in this study.

Study	Country	Participants^*^	Age	BMI, Kg/m^2^	Diabetes duration, yr	Anti-diabetic medications or therapies when they were studied	Lipid-lowering medications when they were studied	Study Design	Anthocyanins source	Dosage	Intervention	Length of intervention	Measured outcomes
Chan et al. (48)	China	20	55.8 ± 9.5	27.2 ± 4.9	n/r	Receiving anti-diabetic medications, but no insulin therapy.	No	Crossover RCT, double-blinded	Bilberry supplementation	~1,400 mg anthocyanins	Patients received either bilberry supplementation daily for 4 weeks, followed by 6 weeks of washout and then an additional 4 weeks of matching placebo or vice versa.	4 weeks	FBG, HbA1c, blood pressure, LDL-C, HDL-C, TC, and TG
Dashti et al. (9)	Iran	C: 21F:19M	C: 47.1 ± 8.21	C: 29.77 ± 4.7	C: 5.25 ± 3.61	Receiving anti-diabetic medications, but no insulin therapy.	No	Parallel RCT, double-blinded	*Ribes khorassanicum* hydro-ethanolic extract	700 mg	The intervention group received extract capsules whereas the control group received a placebo in addition to their regular medication for 3 months.	3 months	FBG, HbA1c, 2 h postprandial glucose, blood pressure, TG, TC, LDL, HDL
		T: 25F:15M	T: 49.07 ± 7.12	T: 29.08 ± 3.58	T: 6.7 ± 4.9								
Fukuda et al. (55)	Japan	C: 19F:20M	C: 62.9 ± 7.8	C:25.0 ± 3.5	C: 9.8 ± 6.2	Receiving anti-diabetic medications or glucagon-like peptide-1 receptor agonists, but no insulin therapy.	No	Parallel RCT, double-blinded	Brazilian green propolis	226.8 mg	The propolis group received Brazilian green propolis (226.8 mg), whereas the placebo group received tablets containing safflower oil, wheat germ oil, and perilla oil.	8 weeks	HOMA-IR, FBG, HbA_1c_, serum insulin, TC, HDL, HDL, TG, remnant-like particle lipoprotein cholesterol, uric acid, Egfr, TNF-α, IL-6, hsCRP, urine Ph and UAE.
		T: 27F:14M	T: 63.7 ± 9.3	T: 25.0 ± 4.8	T: 13.7 ± 10.3								
Kianbakht et al. (49)	Iran	C: 21 M:16F	C: 53.6 ± 8.9	C: 28.9 ± 8.4	C: 8.5 ± 3.8	Receiving anti-diabetic medications, but no insulin therapy.	No	Parallel RCT, double-blinded	Whortleberry fruit extract	1,050 mg	The patients were randomly assigned to be treated with the whortleberry fruit hydroalcoholic extract or placebo	2 months	FBG, 2-h postprandial glucose, HbA1c, and liver/kidney function
		T: 18 M:19F	T: 56.6 ± 10.1	T: 29.3 ± 7.6	T: 7.1 ± 5.4								
Lee et al. (56)	China	C: 7 M: 8F	C: 66 ± 2	C: 25.9 ± 1.0	C: 12 ± 3	Receiving anti-diabetic medications, but no insulin therapy.	No	Parallel RCT, double-blinded	Cranberry extracts	1,500 mg	The patients were randomly assigned to receive cranberry extracts or placebo for 12 weeks.	12 weeks	Changes in lipid profile, oxidized LDL, glycaemic control, components of the metabolic syndrome, C-reactive protein (CRP), and urinary albumin excretion
		T: 9 M: 6F	T: 65 ± 2	T: 26.2 ± 0.7	T: 8 ± 1								
Li et al. (50)	China	C: 17 M:12F	C: 57.6 ± 3.4	C: 25.3 ± 2.5	n/r	Around half of the participants were receiving oral anti-diabetic drugs. 7 of the participants were receiving insulin therapy.	Only 4 of the participants were taking lipid-lowering drugs.	Parallel RCT, double-blinded	Purified anthocyanin supplementation	~320 mg anthocyanins	T2DM patients were randomly assigned to receive 160 mg of anthocyanins twice daily or a placebo (n = 29/group) for 24 wk	6 months	Serum adipokine, proinflammatory molecules, glycemic indexes, lipid profiles, and antioxidant capacity
		T: 17 M:12F	T: 58.1 ± 2.3	T: 25.1 ± 2.7									
Moazen et al. (59)	Iran	C: 17	C: 51.17 ± 13.88	C: 28.70 ± 4.24	At least 1 year	n/r	No	Parallel RCT, double-blinded	Freeze-dried strawberries	~154 mg anthocyanins	In all, 36 subjects with T2D were randomly divided into two groups. The treatment group consumed two cups of FDS beverage or macronutrient-matched placebo powder with strawberry flavor daily for 6 weeks.	6 weeks	FBG, HbA1c, and other metabolic biomarkers
		T: 19	T: 51.88 ± 8.26	T: 27.32 ± 3.26									
Schell et al. (51)	USA	5 M:20F	54 ± 4.2	35.3 ± 2.0	At least 5 years	60% of the participants were receiving oral anti-diabetic drugs. No one was under insulin therapy.	No	Crossover RCT	Raspberry supplement	~225 mg anthocyanins	Two different phases: a “postprandial phase” of acute raspberry supplementation (2 separate days at least 1 week apart), followed by a 1-week washout phase, and then a 10-week “diet supplement phase,” with and without raspberry supplementation periods of 4 weeks each, separated by 2-week washout phase.	18 weeks	Glycemic markers and inflammatory profile
Shidfar et al. (57)	Iran	C: 29	54.8 ± 9.1	C: 28.4 ± 2.4	Less than 5 years	Receiving anti-diabetic drugs, but not insulin.	No	Parallel RCT, double-blinded	Cranberry juice	~0.672 mg	Patients with T2DM were randomly assigned to receive either one cup of cranberry juice (CJ) or a placebo drink daily for 12 weeks	12 weeks	FBG, serum glucose, PON-1 activity, ApoB, apoA-I, and Lp(a) were determined.
		T: 29		T: 29.1 ± 2.8									
Sohrab et al. (60)	Iran	C: 30	C: 55.3 ± 8.5	C: 26.5 ± 3.6	At least 1 year	Receiving or not receiving anti-diabetic drugs and not taking insulin therapy.	No	Parallel RCT, single-blinded	Pomegranate juice	~0.842 mg anthocyanins	A total of 60 patients with diabetes were given 200 mL of pomegranate juice daily or placebo (*n* = 30/group) for 6 weeks.	6 weeks	FBG, ox-LDL, anti-ox-LDL, total antioxidant capacity, PON1
		T: 30	T: 54.6 ± 8.4	T: 27.2 ± 3.4									
Soltani et al. (52)	Iran	C: 19 M:11F	C: 49.93 ± 6.12	C: 29.21 ± 2.01	At least 2 years	Receiving anti-diabetic drugs. Not using any insulin preparation and/or any antidiabetic drug increases endogenous insulin secretion.	No	Parallel RCT, double-blinded	Cornelian cherry (*Cornus mas* L.) extract	~300 mg anthocyanins	Patients with T2DM were randomly assigned to two groups to receive either the extract or placebo capsules for 6 weeks	6 weeks	FBG, insulin, HbA_1C_, and TG as well as 2-h postprandial glucose level
		T: 20 M:10F	T: 49.16 ± 5.62	T: 29.40 ± 1.73									
Stote et al. (53)	Canada	52 M	C: 66.7 ± 1.1	C: 34.0 ± 0.9	At least 6 months	Receiving anti-diabetic drugs and not taking insulin therapy.	65% of the intervention group and 88% of the control group on lipid-lowering medication (HMG-CoA reductase inhibitors).	Parallel RCT, double-blinded	Freeze-dried blueberries	~261.8 mg anthocyanins	Patients with T2DM were randomly assigned to one of two intervention groups, with either 22 g freeze-dried blueberries or 22 g placebo.	8 weeks	Glycemic markers
			T: 67.1 ± 1.1	T: 34.2 ± 0.7									
Yang et al. (54)	China	C: 31	C: 61.2 ± 6.9	C: 24.8 ± 3.4	Newly diagnosed diabetes	Not taking any diabetic medications or therapies.	No	Parallel RCT	Anthocyanins supplement	~320 mg anthocyanins	Newly diagnosed patients with T2DM were randomly assigned to either the anthocyanins group or the placebo group for 12 weeks	12 weeks	Serum adiponectin, biomarkers related to glucolipid metabolism, anthropometric parameters, dietary intake, and physical activity
		T: 39	T: 60.8 ± 7.9	T: 24.7 ± 3.2									

^*^C, control group; T, treatment group; The dosage of the study done by Shidfar et al. (57) was estimated by Duthie et al. (61). The dosage of the study done by Sohrab et al. (60) was estimated by Trishitman et al. (62).

**Table 3:** Baseline of the main investigated parameters in the included studies.

Study	Baseline fasting blood glucose, mmol/L^*^	Baseline HbA_1c_, %	Baseline triglycerides, mmol/L	Baseline total cholesterol, mmol/L	Baseline HDL, mmol/L	Baseline LDL, mmol/L
Chan et al. (48)	C: 8.66 ± 1.82	C: 7.58 ± 1.26	C: 1.86 ± 1.47	C: 4.90 ± 0.75	C: 1.35 ± 0.49	C: 2.72 ± 0.49
	T: 8.75 ± 2.87	T: 7.37 ± 1.06	T: 1.78 ± 1.21	T: 5.01 ± 0.77	T: 1.42 ± 0.52	T: 2.85 ± 0.76
Dashti et al. (9)	C: 9.64 ± 2.31	C: 8.24 ± 1.14	C: 9.19 ± 3.56	C: 4.34 ± 1.84	C: 2.16 ± 0.31	C: 6.14 ± 1.89
	T: 9.86 ± 2.21	T: 8.20 ± 1.31	T: 8.92 ± 3.89	T: 4.75 ± 2.17	T: 2.10 ± 0.36	T: 6.36 ± 1.97
Fukuda et al. (55)	C: 7.70 ± 1.86	C: 7.21 ± 0.85	C: 1.82 ± 1.07	C: 10.08 ± 1.94	C: 3.02 ± 0.91	C: 6.36 ± 1.62
	T: 7.43 ± 1.40	T: 7.09 ± 0.79	T: 1.84 ± 1.05	T: 10.01 ± 1.63	T: 3.31 ± 0.84	T: 6.02 ± 1.34
Kianbakht et al. (49)	C: 9.98 ± 1.90	C: 7.3 ± 1.1	n/r	n/r	n/r	n/r
	T: 10.64 ± 1.49	T: 7.6 ± 0.9				
Lee et al. (56)	C: 8.3 ± 0.4	C: 8.0 ± 0.2	C: 1.6 ± 0.1	C: 5.1 ± 0.2	C: 1.3 ± 0.1	C: 3.0 ± 0.1
	T: 8.9 ± 0.5	T: 8.1 ± 0.2	T: 1.8 ± 0.2	T: 5.3 ± 0.2	T: 1.3 ± 0.1	T: 3.3 ± 0.2
Li et al. (50)	C: 7.3 ± 1.7	C: 6.5 ± 1.4	C: 2.02 ± 0.36	C: 5.03 ± 0.78	C: 0.98 ± 0.08	C: 3.19 ± 0.42
	T: 7.1 ± 2.2	T: 6.2 ± 1.9	T: 2.04 ± 0.41	T: 5.07 ± 0.89	T: 1.03 ± 0.11	T: 3.17 ± 0.35
Moazen et al. (59)	C: 11.2 ± 5.0	C: 7.46 ± 1.9	n/r	n/r	n/r	n/r
	T: 8.9 ± 2.8	T: 7.23 ± 1.6				
Schell et al. (51)	7.88 ± 0.61	n/r	8.99 ± 0.83	10.66 ± 0.61	2.50 ± 0.14	6.38 ± 0.61
Shidfar et al. (57)	C: 7.58 ± 0.51	n/r	n/r	n/r	n/r	n/r
	T: 7.74 ± 0.52					
Sohrab et al. (60)	C: 10.07 ± 3.5	n/r	n/r	n/r	n/r	n/r
	T: 8.8 ± 2.3					
Soltani et al. (52)	C: 9.69 ± 2.26	n/r	C: 13.18 ± 8.27	n/r	n/r	n/r
	T: 8.77 ± 2.30		T: 11.01 ± 3.70			
Stote et al. (53)	C:8.37 ± 0.26	C: 7.4 ± 0.2	C: 9.79 ± 0.85	C: 9.24 ± 0.53	C: 2.70 ± 0.14	C: 4.84 ± 0.47
	T: 8.24 ± 0.27	T: 7.2 ± 0.2	T: 10.33 ± 1.37	T: 8.96 ± 0.53	T: 2.48 ± 0.11	T: 4.60 ± 0.43
Yang et al. (54)	C: 6.75 ± 0.68	C: 6.45 ± 0.52	C: 1.92 ± 1.74	C: 6.14 ± 1.53	C: 1.45 ± 0.35	C: 3.35 ± 1.07
	T: 6.42 ± 0.71	T: 6.15 ± 0.41	T: 1.77 ± 1.20	T: 6.21 ± 1.24	T: 1.48 ± 0.37	T: 3.39 ± 0.95

^*^C, control group; T, treatment group.

### 3.3. Risk of bias of selected RCTs

Individual Cochrane Risk of Bias assessment has been presented in Figure 1C for each included RCT. The findings indicate that all included RCTs had a low risk of bias for random sequence generation, blinding (performance bias and detection bias), incomplete outcome data, and selective reporting. More than half (69.2%) of the RCTs had an unclear risk of bias for allocation concealment as the authors did not report this in the articles (9, 48, 50, 51, 54–57, 59). Other studies exhibited a low risk of allocation concealment (49, 52, 53, 60). The majority of the RCTs exhibited a low risk of blinding bias for participants and personnel and detection of outcome assessment (9, 48–50, 52, 53, 55–57, 59), except three of them had an obvious serious risk of blinding bias (51, 54, 60). All the RCTs were graded to have an unclear or low risk of other bias.

### 3.4. The impact of anthocyanins on glycemic parameters and insulin resistance

Anthocyanins have been reported to exert positive effects on glycemic regulation as well as insulin resistance due to their antioxidant and anti-inflammatory capacities, by different research studies. In order to have a better understanding of the degree of the beneficial effects in patients with T2DM, we systematically evaluated the findings on the effect of anthocyanins on specific glycemic and insulin resistance markers including FBG, HbA_1c_, 2-h postprandial blood glucose, fasting insulin, and HOMA-IR.

#### 3.4.1. Impact of anthocyanins on HbA_1c_

**Figure 2:**
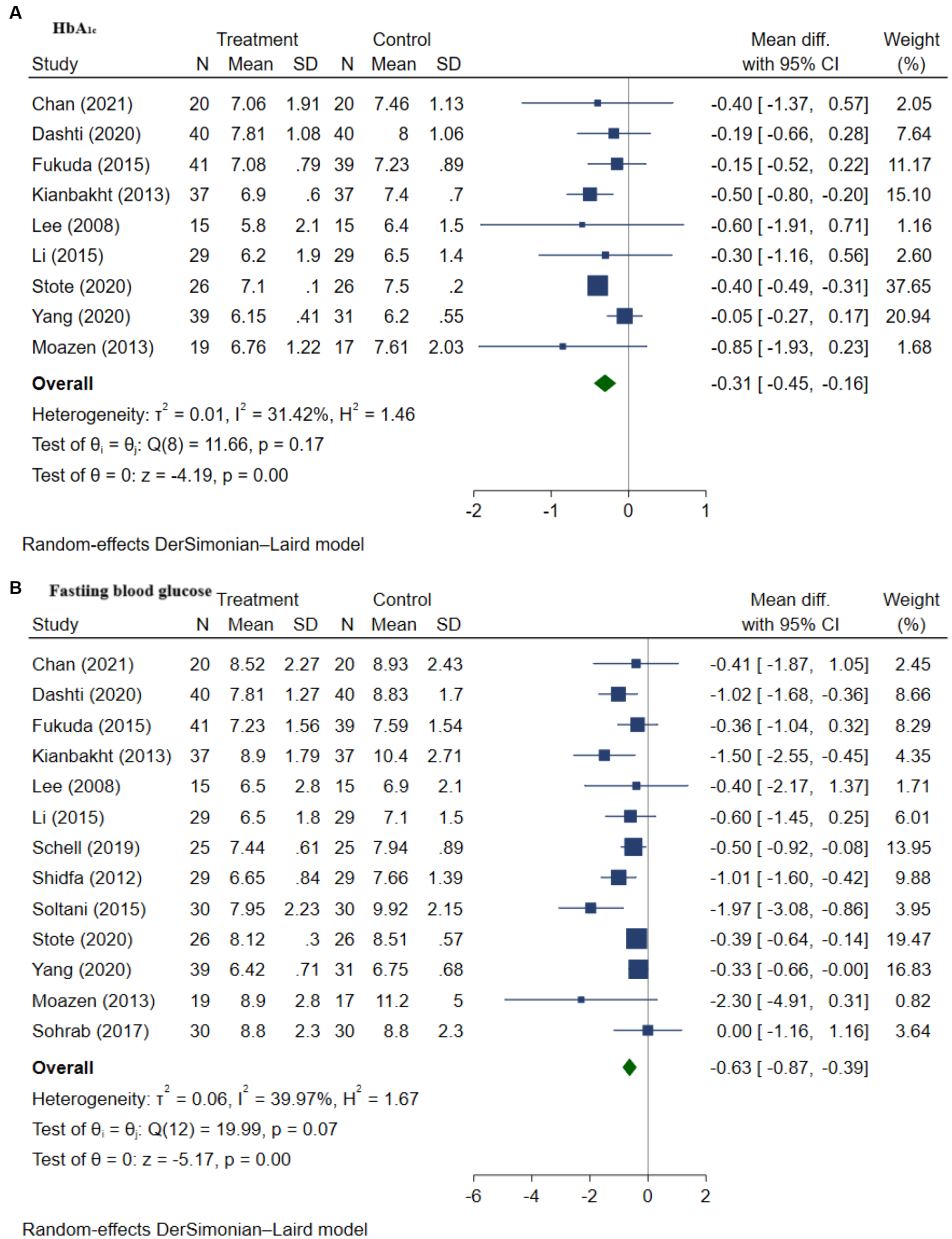

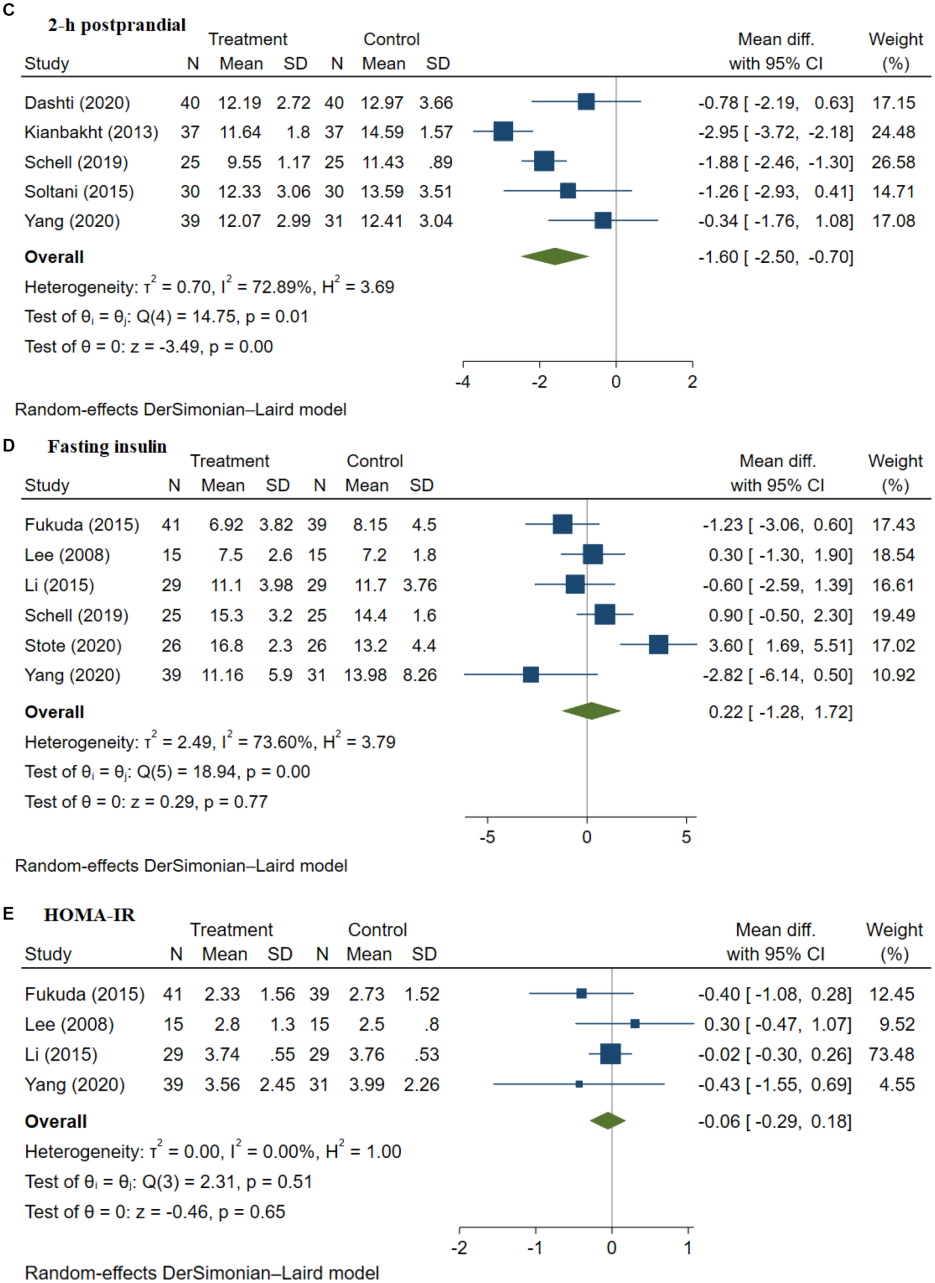
**(A–E)** Meta-analysis of the effect of anthocyanins on HbA_1c_ **(A)**, fasting blood glucose **(B)**, 2-h postprandial glucose **(C)**, fasting insulin **(D)**, and HOMA-IR **(E)** in patients with type 2 diabetes. Diamonds represent the pooled effect estimate for overall analysis. Data were represented as the mean difference with 95% CI, using the random-effects DerSimonian-Laird model. Between-study heterogeneity quantified by *I*^2^ with significant *p* < 0.05. Test of homogeneity (θ_i_ = θ_j_) of study-specific effects sizes, with the chi-squared test statistic rejected if *p* < 0.05.

The impact of anthocyanins on HbA_1c_ in patients with T2DM was assessed in nine RCTs comprising 500 participants (9, 48–50, 53–56, 59). In the anthocyanin intervention group, HbA_1c_ was substantially reduced by 0.31% ([95% CI -0.45, −0.16], *p* = 0.00; Figure 2A) when compared to the placebo group. No substantial between-study heterogeneity was identified (I^2^ = 31.42%, *p* = 0.17), corresponding to the results found by the Galbraith plot test. Further subgroup analysis showed no significant difference between intervention length ≥ 8 weeks and intervention difference < 8 weeks regarding the effect in reducing HbA_1c_ (*p* = 0.41; Supplementary Figure 1A). However, subgroup analysis of different dosage groups (≥320 mg anthocyanins equivalent, <320 mg anthocyanins equivalent, unknown anthocyanins amount equivalent; *p* = 0.02; Supplementary Figure 1B) revealed that a dosage ≥ 320 mg anthocyanins contributed to heterogeneity and had the least effect size. Besides, anthocyanins from fruit extract/powder showed a significantly higher effect in reducing HbA_1c_ in patients with T2DM (*p =* 0.01; Supplementary Figure 1C). Publication bias was not revealed according to Egger’s regression test (*p* = 0.624) for studies evaluating HbA_1c_. Besides, further trim-and-fill analysis also did not identify any missing studies.

#### 3.4.2. Impact of anthocyanins on fasting blood glucose

The impact of anthocyanins on fasting blood glucose (FBG) was evaluated in 13 RCTs involving 703 T2DM patients (9, 48–57, 59, 60). Our results indicate that a median dose of 320 mg anthocyanins per day for a median intervention length of 8 weeks resulted in a substantial drop in FBG compared to placebo groups, with a WMD of −0.63 mmol/L ([95% CI −0.87, −0.39], *p* = 0.00; Figure 2B). All of the studies reported a reduction in FBG value after the intervention with pure anthocyanin supplements or foods with anthocyanins.

No significant between-study heterogeneity was detected in the analysis (I^2^ = 39.97%, *p* = 0.07), however, heterogeneity analysis by Galbraith plot found that the study conducted by Soltani et al. (52) was a source of heterogeneity. After removing the trial, the overall heterogeneity was substantially reduced (I^2^ = 17.49%, *p* = 0.27) and the modified effect size was a little bit lower compared to the previous result, which was −0.54 mmol/L with a 95% CI of −0.73 to −0.35 mmol/L and maintained significant (*p* = 0.00; Supplementary Figure 2A). Subgroup analysis without including the study indicating heterogeneity revealed no significant results between groups with an intervention length greater than 8 weeks and length less than 8 weeks (Supplementary Figure 2B). Moreover, no significant difference was observed between anthocyanins source groups (fruit extract/powder and pure anthocyanins; *p* = 0.22; Supplementary Figure 2C), as well as among intervention dosage groups (≥320 mg anthocyanins equivalent, <320 mg anthocyanins equivalent, unknown anthocyanins amount equivalent; *p* = 0.42; Supplementary Figure 2D). Funnel plot (Supplementary Figure 2E) and Egger’s test (*p* = 0.013) demonstrated publication bias and a trim-and-fill test confirmed one missing study, with the WMD declining after filling the missing study (WMD −0.62 mmol/L [95% CI -0.87, −0.38]).

#### 3.4.3. Impact of anthocyanins on 2-h postprandial glucose

The impact of anthocyanins on 2-h postprandial glucose level in 309 patients with T2DM was investigated in five studies (9, 49, 51, 52, 54); a reduction in 2-h postprandial glucose level (WMD −1.60 [95% CI −2.50, −0.70], *p* = 0.00; Figure 2C) was noted with anthocyanins intervention. All of the included studies demonstrated a reduction in 2-h postprandial glucose level after intervention with anthocyanins in patients with T2DM.

There was substantial between-study heterogeneity of I^2^ = 72.89% (*p* = 0.01). Galbraith plot test found that the studies conducted by Kianbakht et al. (49) and Yang et al. (54) were the sources of heterogeneity. Further leave-one-out sensitivity analyses stated that the between-study heterogeneity dropped (I^2^ = 43.35%, *p* = 0.15) after the omission of the study done by Kianbakht et al. (49), and the effect size changed to −1.25 mmol/L ([95% CI −2.04, −0.47], *p* = 0.00; Supplementary Figure 3). Egger’s regression test failed to uncover any evidence of publication bias (*p* = 0.093), whereas trim-and-fill analysis revealed that there were two missing studies. After accounting for these missing studies, the overall effect changed to −2.15 mmol/L [95% CI −3.03, −1.26].

#### 3.4.4. Impact of anthocyanins on fasting insulin and HOMA-IR

The impact of anthocyanins on fasting insulin and HOMA-IR in patients with T2DM was evaluated in six studies involving 315 patients with T2DM (50, 51, 53–56) and four studies involving 238 patients with T2DM (50, 54–56), respectively. No significant result was found in both fasting insulin level (WMD 0.22 [95% CI −1.28, 1.72], *p* = 0.77, Figure 2D) and HOMA-IR level (WMD −0.06 [95% CI -0.29, 0.18], *p* = 0.65, Figure 2E). A considerable between-study heterogeneity was discovered in evaluating the effect of anthocyanins in fasting insulin (I^2^ = 73.60%, *p* = 0.00). No cross-study heterogeneity was found for HOMA-IR (I^2^ = 0.00%, *p* = 0.51), although it was not statistically significant. Publication bias was found in fasting insulin level (*p* = 0.028), however, no publication bias was found in HOMA-IR (*p* = 0.388). The further non-parametric trim-and-fill analysis found one missing study for fasting insulin, which was then imputed, and the mean difference increased to 0.60 mU/L. Trim-and-fill analysis suggested one missing study, on imputing this study, the overall effect on HOMA-IR adjusted to −0.04.

Overall, the intervention of anthocyanins significantly decreased FBG value by 0.64 mmol/L, HbA_1c_ by 0.29%, and 2-h postprandial glucose value by 1.60 mmol/L in patients with T2DM. However, the results were insignificant regarding fasting insulin and HOMA-IR.

### 3.5. Impact of anthocyanins on lipid profile

Patients with T2DM are particularly vulnerable to dyslipidemia, which is closely linked to the development of cardiovascular diseases, the primary comorbidity in patients with T2DM (63). This is related to chronic inflammation due to long-term hyperglycemia. Anthocyanins have been found to be effective as anti-inflammatory and as antioxidative bioactive compounds, however, it is unclear to what extent they affect the lipid profile of patients with T2DM. The only available study on this aspect has included multiple types of participants in the analysis such as healthy people and patients with cardiovascular diseases (24). Thus far, no studies have addressed the effects of anthocyanins in patients with T2DM only. In addition, our study is the only study evaluating the impact of anthocyanins on lipid profile (including TG, TC, HDL cholesterol, and LDL cholesterol) in patients with T2DM.

#### 3.5.1. Impact of anthocyanins on triglycerides

**Figure 3:**
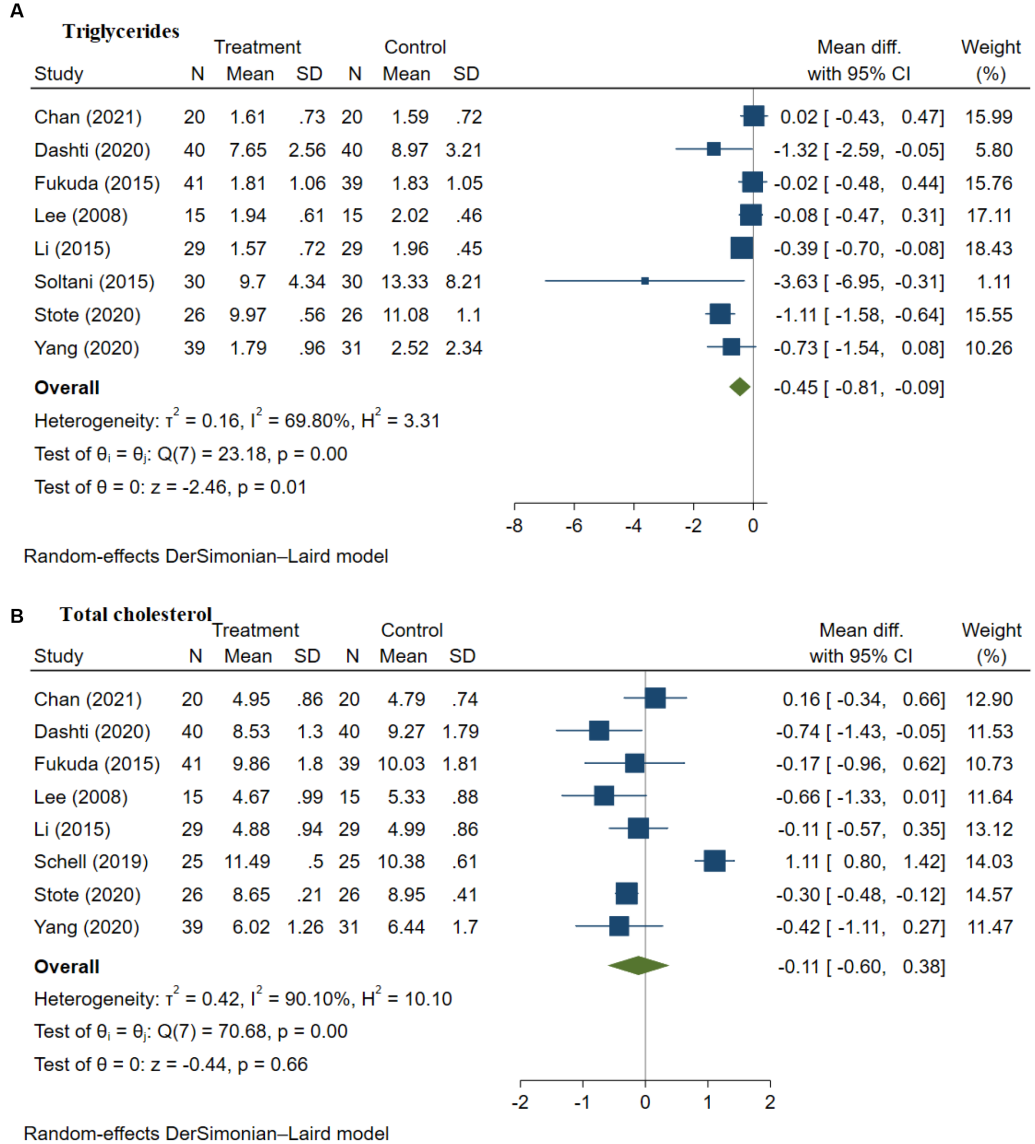

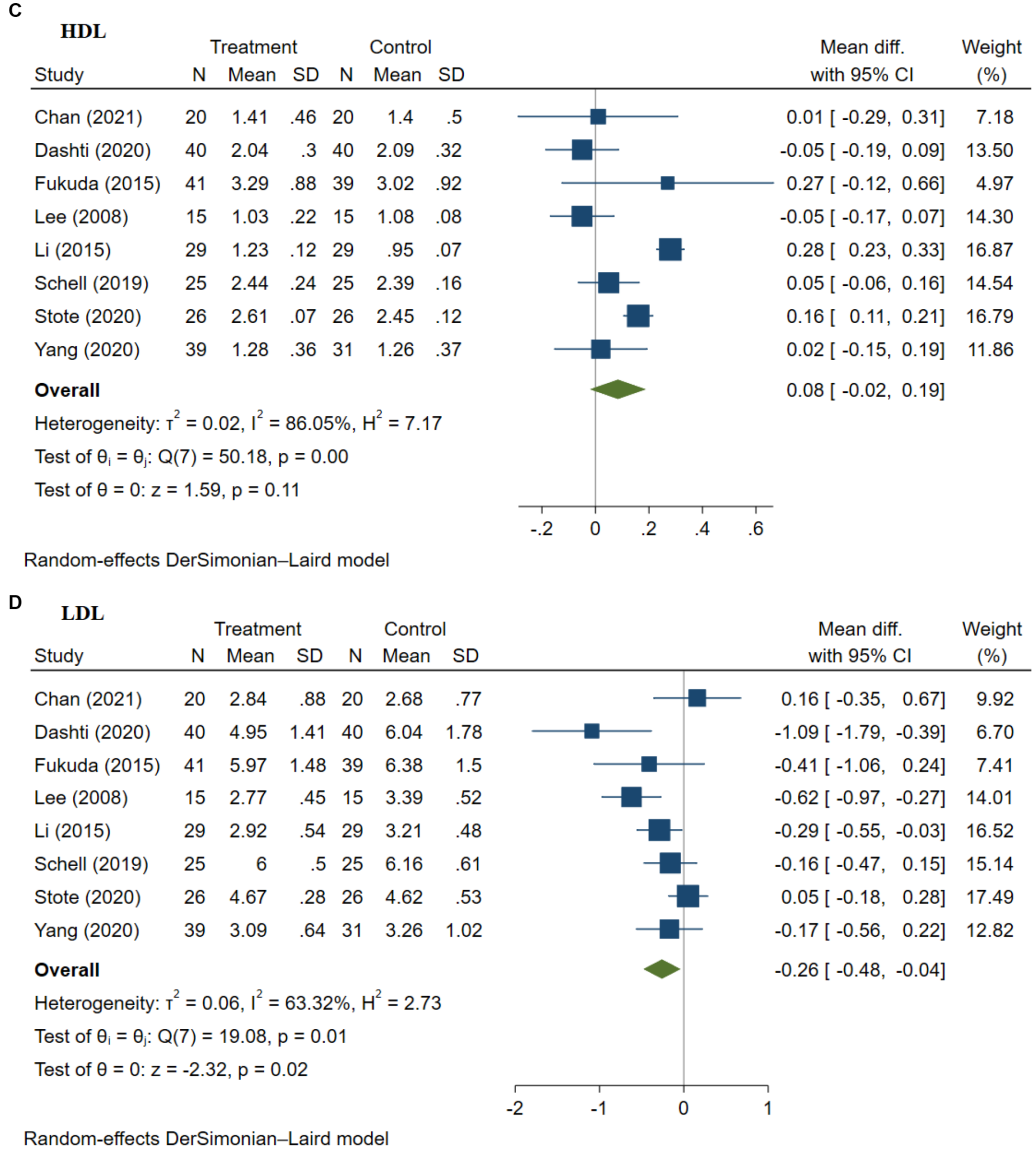
**(A–D)** Meta-analysis of the effect of anthocyanins on lipid profile – triglycerides (TG, **A**), total cholesterol (TC, **B**), high-density lipoprotein cholesterol (HDL, **C**), and low-density lipoprotein cholesterol (LDL, **D**) in patients with type 2 diabetes. Diamonds represent the pooled effect estimate for overall analysis. Data were represented as the mean difference with 95% CI, using the random-effects DerSimonian-Laird model. Between-study heterogeneity quantified by *I*^2^ with significant *p* < 0.05. Test of homogeneity (θ_i_ = θ_j_) of study-specific effects sizes, with the Chi-squared test statistic rejected if *p* < 0.05.

The impact of anthocyanins on TG was assessed in six studies with a total of 450 T2DM patients (9, 48, 50, 52–56). A remarkable reduction in TG was detected in the anthocyanins intervention group as compared to the placebo group, with an effect size of −0.45 mmol/L ([95% CI −0.81, −0.09], *p* = 0.01; Figure 3A). There was notable between-study heterogeneity (I^2^ = 69.80%, *p* = 0.00). Further heterogeneity analysis by the Galbraith plot indicated that the study by Stote et al. (53) was the main reason for heterogeneity. After removing the study, the heterogeneity greatly decreased, with I^2^ = 49.57% (*p* = 0.06; Supplementary Figure 4). The effect size was modified, with a WMD of −0.28 mmol/L ([95% CI −0.58, 0.02], *p* = 0.07). Publication bias was observed (*p* = 0.01). One missing trial was found by additional trim-and-fill analysis. After imputing the study, the effect size was adjusted to −0.42 mmol/L, with a 95% CI of −0.79 to −0.04.

#### 3.5.2. Impact of anthocyanins on total cholesterol

The impact of anthocyanins on total cholesterol (TC) was analyzed in six studies with 415 patients with T2DM (9, 48, 50, 51, 53–56). There was no statistical difference in TC (WMD −0.11 [95% CI −0.61, 0.38], *p* = 0.66; Figure 3B), and large between-study heterogeneity was observed (I^2^ = 90.10%, *p* = 0.00). The leave-one-out sensitivity analysis stated that after excluding Schell et al. (51) from the analysis, the between-study heterogeneity dropped sharply (I^2^ = 11.35%, *p* = 0.34) with a WMD of −0.28 mmol/L ([95% CI -0.45, −0.01], *p* = 0.00; Supplementary Figure 5A). The subgroup analysis based on the source of anthocyanins also explained the heterogeneity (Supplementary Figure 5B), as between-study heterogeneity was only found in the studies using anthocyanins from fruit extracts. Intervention with pure anthocyanins supplements (I^2^ = 0.00%, *p* = 0.47) decreased total cholesterol by 0.21 mmol/L, to a greater extent compared to the intervention using anthocyanins from fruit extracts (I^2^ = 92.78%, *p* = 0.00), with a reduction of 0.07 mmol/L. Publication bias was found, with (*p* = 0.00) subsequent trim-and-fill analysis disclosing two missing studies. After including the two missing studies, the impact size was adjusted to 0.072 [95% CI −0.343, 0.487].

#### 3.5.3. Impact of anthocyanins on HDL cholesterol

The impact of anthocyanins on HDL was explored in six studies in 415 patients with T2DM (9, 48, 50, 51, 53–56) and no significant results were observed (WMD 0.08 [95% CI −0.02, 0.19], *p* = 0.11; Figure 3C). Besides, there was substantial between-study heterogeneity (I^2^ = 86.05%, *p* = 0.00). The subsequent leave-one-out sensitivity analysis observed that the studies by Dashti et al. (9), Lee et al. (56), and Li et al. (50) could explain the heterogeneity. After the removal of these studies, the heterogeneity decreased, with I^2^ = 31.23% (*p* = 0.21). The modified effect of anthocyanin intervention on blood HDL level was 0.11 mmol/L ([95% CI 0.03, 0.18], *p* = 0.01; Supplementary Figure 6). Publication bias was found (*p* = 0.003), however, no missing study was identified by further trim-and-fill analysis.

#### 3.5.4. Impact of anthocyanins on LDL cholesterol

The impact of anthocyanins on LDL was determined in six studies in 415 patients with T2DM (9, 48, 50, 51, 53–56), and a decrease in LDL level was found (WMD −0.26 [95% CI −0.48, −0.04], *p* = 0.02; Figure 3D). There was between-study heterogeneity (I^2^ = 63.32%, *p* = 0.01), and the studies by Dashti et al. (9) and Stote et al. (53) were found to be the source of heterogeneity according to the Galbraith plot. After removing these studies, the heterogeneity decreased substantially (I^2^ = 33.56%, *p* = 0.18), with an overall effect remaining the same (WMD −0.26 [95% CI −0.45, −0.08], *p* = 0.01). No missing study was noticed, despite publication bias being discovered (*p* = 0.012).

Overall, our results found that anthocyanins, either from fruit extracts or supplements, significantly reduced TG and LDL, with an absolute reduction of 0.45 mmol/L and 0.26 mmol/L, respectively. However, no significant results were found regarding TC and HDL levels. Further analysis indicated that pure anthocyanin supplements exhibited a higher reduction in TC and improvement in HDL cholesterol than fruit extracts. There is a need for more well-designed long-term RCTs to figure out the impact of anthocyanin supplements in the diet on the blood lipid profile.

### 3.6. Impact of anthocyanins on blood pressure

There is a high prevalence of hypertension among patients with T2DM compared to those without diabetes, affecting more than 73.6% of patients with T2DM in the USA according to the National Diabetes Statistics Report (2020) revealed by the Centers for Disease Control and Prevention. Diabetes-induced chronic hyperglycemic and insulin resistance leads to increased vascular oxidative stress, inflammation, and endothelial dysfunction, which in turn promote vascular stiffness or aging, resulting in an increase in blood pressure and the onset of cardiovascular disease (64, 65). Therefore, control of blood pressure in patients with T2DM is critically important to delay the onset of other complications. Conflicting results have been reported regarding the effectiveness of anthocyanins on blood pressure by different research studies (48, 53, 56), and hence, we have evaluated the impact of anthocyanins on T2DM patients’ blood pressure, which is a marker for cardiovascular health.

#### 3.6.1. Impact of anthocyanins on systolic blood pressure

**Figure 4:**
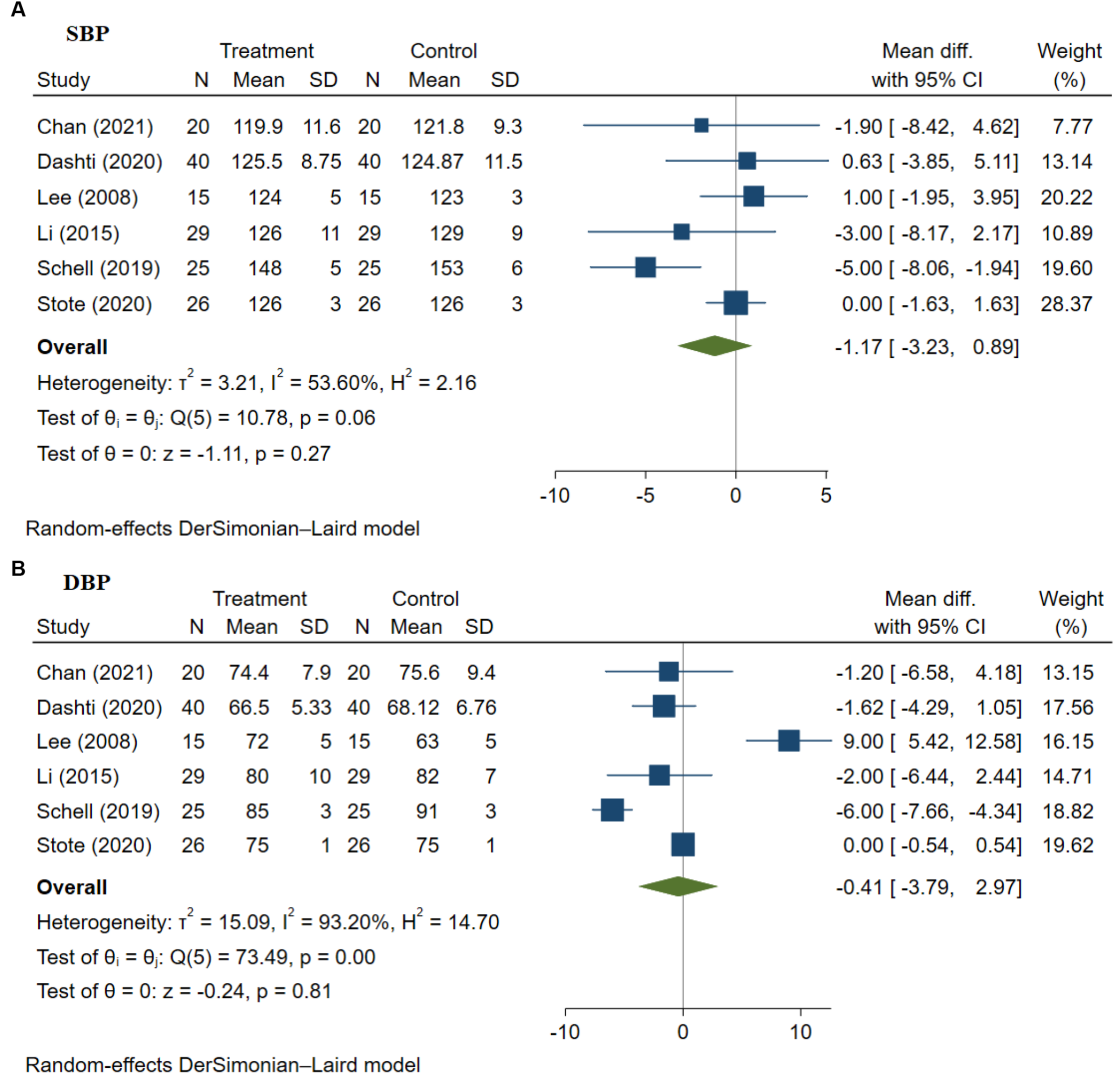
**(A,B)** Meta-analysis of the effect of anthocyanins on blood pressure—systolic blood pressure **(A)** and diastolic blood pressure **(B)** in patients with type 2 diabetes. Diamonds represent the pooled effect estimate for overall analysis. Data were represented as the mean difference with 95% CI, using the random-effects DerSimonian-Laird model. Between-study heterogeneity quantified by *I*^2^ with significant *p* < 0.05. Test of homogeneity (θ_i_ = θ_j_) of study-specific effects sizes, with the Chi-squared test statistic rejected if *p* < 0.05.

The impact of anthocyanins on systolic blood pressure was tested in six studies with a total of 265 patients with T2DM (9, 48, 50, 51, 53, 56). The effect of anthocyanins on systolic blood pressure was not statistically significant (WMD −1.17 [95% CI −3.23, 0.89], *p* = 0.27; Figure 4A). Besides, there was between-study heterogeneity (I^2^ = 53.60%, *p* = 0.06) and the study by Schell et al. (51) was found to be responsible for the heterogeneity. The removal of this study from the analysis decreased the heterogeneity (I^2^ = 0.00%, *p* = 0.71), however, no statistical significance was found in the decline in the systolic blood pressure with the anthocyanin intervention (*p* = 0.98). Egger’s test revealed no evidence of publication bias (*p* = 0.054), and subsequent trim-and-fill analysis indicated no evidence of missing studies.

#### 3.6.2. Impact of anthocyanins on diastolic blood pressure

The impact of anthocyanins on diastolic blood pressure was analyzed in six studies with 265 patients with T2DM (9, 48, 50, 51, 53, 56). No significant result was found in diastolic blood pressure (WMD −0.41 [95% CI −3.79, 2.97], *p* = 0.81; Figure 4B) and there was substantial between-study heterogeneity (I^2^ = 93.20%, *p* = 0.00). Further leave-one-out sensitivity analysis found that the studies by Lee et al. (56) and Schell et al. (51) were the explanation of heterogeneity. After omitting these studies, the cross-study heterogeneity dropped sharply, with I^2^ = 0.00% (*p* = 0.52, Supplementary Figure 7) but the result was still not significant (*p* = 0.70). Publication bias was discovered according to Egger’s test (*p* = 0.003) and subsequent trim-and-fill analysis found two missing studies. After including the two missing studies, the impact size was adjusted to 1.524 [95% CI −2.220, 5.268].

Overall, no significant effects were found regarding the impact of anthocyanin intervention on human blood pressure in the clinical trials evaluated. More long-term RCTs are needed to further investigate the effect of anthocyanins on human blood pressure.

### 3.7. Grading of the evidence

The evidence of anthocyanins significantly reducing the glycemic indexes (FBG, HbA_1c_, and 2-h postprandial glucose) and lipid profile (TG and LDL cholesterol) was graded according to GRADE Criteria and summarized in Table 4. Although publication bias was detected, evidence for FBG was of large effect size and thus rated as high quality in terms of grade. The effects estimated for HbA_1c_ were also rated as high quality as no downgrade was observed of inconsistency, imprecision, or publication bias. The evidence for 2-h postprandial glucose was rated as moderate as inconsistency was found to be serious. The evidence for TG and LDL cholesterol were also rated as moderate owing to the inconsistency reported. However, we did not grade the evidence for fasting insulin, HOMA-IR, TC, HDL, SBP, and DBP as the results for these outcomes were not significant. There is a need for more RCTs studying these parameters that were not evaluated to figure out the impact of anthocyanins on these parameters in patients with T2DM and related cardiovascular diseases.

**Table 4:** Critical and important evidence for the different markers in randomized trials (RTs) evaluated based on GRADE criteria.

Certainty assessment							No of patients		Effect	Certainty	**Importance**
No of studies	Study design	Risk of bias	Inconsistency	Indirectness	Imprecision	Other considerations	Fasting blood glucose	placebo	Absolute (95% CI)		
**Fasting blood glucose**											
11	RTs	Not serious	Not serious	Not serious	Not serious	Publication bias is strongly suspected strong association^a^	380	368	MD**0.63 lower** (0.87 lower to 0.39 lower)	⨁⨁⨁⨁	CRITICAL
										High	
**HbA**_**1c**_											
8	RTs	Not serious	Not serious	Not serious	Not serious	None	266	254	MD**0.31 lower** (0.45 lower to 0.16 lower)	⨁⨁⨁⨁	CRITICAL
										High	
**2-h postprandial glucose**											
5	RTs	Not serious	Serious^b^	Not serious	Not serious	None^c^	171	163	MD**1.6 lower** (2.5 lower to 0.7 lower)	⨁⨁⨁◯	IMPORTANT
										Moderate	
**TG**											
8	RTs	Not serious	Serious^d^	Not serious	Not serious	Publication bias is strongly suspected strong association^e^	240	230	MD**0.45 lower** (0.81 lower to 0.09 lower)	⨁⨁⨁◯	IMPORTANT
										Moderate	
**LDL**											
8	RTs	Not serious	Serious^f^	Not serious	Not serious	None	235	225	MD**0.26 lower** (0.48 lower to 0.04 lower)	⨁⨁⨁◯	IMPORTANT
										Moderate	

CI, confidence interval; MD, mean difference. ^a^Publication bias was noted by both the funnel plot and Egger’s test (*p* = 0.013). The further trim-and-fill test indicated one missing study. ^b^Evidence of substantial between-study heterogeneity (I^2^ = 72.89%, *p* = 0.01), however, Kianbakht et al. (49) accounted for 29.54% of heterogeneity. ^c^No publication bias was detected by Egger’s regression test (*p* = 0.093). However, further trim-and-fill analysis revealed there were two missing studies. ^d^There was substantial heterogeneity between studies (I^2^ = 69.80%, *p* = 0.00), but it could be explained by leave-one-out sensitivity analysis. ^e^Publication bias was detected, with *p* = 0.01. Further trim-and-fill analysis detected one missing study. ^f^There was between-study heterogeneity (I^2^ = 63.32%, *p* = 0.01), but it could be explained. Parameters rated as critical/important and it’s effect size are bolded.

## 4. Discussion

The current systematic review and meta-analysis involved 13 RCTs including 703 participants and evaluated the impact of anthocyanins on glycemic indicators, insulin resistance, and cardiovascular biomarkers including lipid profile and blood pressure in patients with T2DM. This is the first systematic review and meta-analysis on anthocyanins’ effects on individuals with T2DM and investigating the effects of anthocyanins on blood pressure, as reported by RCTs. Our findings indicate that a median dose of 320 mg/day anthocyanins for a period of 8 weeks significantly reduced the markers of glycemic control in individuals with T2DM including HbA_1c_, FBG, and 2-h postprandial glucose level, with an absolute reduction of 0.31%, 0.63 mmol/L, and 1.6 mmol/L, respectively. This is supported by Neyestani et al. (28), who claimed a significant reduction in FBG (0.85 mmol/L) after administrating anthocyanins in patients with T2DM and a decrease in HbA_1C_ (0.14%) in individuals with and without T2DM. Our findings were also consistent with that observed by a meta-analysis evaluating 32 studies performed with both healthy and cardiometabolic diseased populations on the effect of anthocyanins from fruit extracts or supplements. The meta-analysis also reported significant reductions in HbA_1c_, FBG, and 2-h postprandial glucose, with an average effect of 0.65%, 0.31 mmol/L, and 0.82 mmol/L, respectively (24). Furthermore, Tiwari et al. (27) also reported a significant decrease in HbA_1c_ and FBG by 0.80% and 1.54 mmol/L, respectively, in a systematic review and meta-analysis based on animal studies and human studies in patients with obesity or were overweight, had pre-diabetes, and T2DM.

Our findings from the subgroup analysis indicated that the reduction of HbA_1c_ was found to be dependent on the source of anthocyanins. Anthocyanins from fruit extracts/powder experienced a higher reduction in HbA_1c_ than from pure anthocyanins supplements. It may be related to other phytochemicals or synergistic effects of different phytochemicals contained in the fruit extracts as well as the dosage of anthocyanins, however, details about the other compounds present that could be playing a role are lacking in these publications. On the other hand, the intervention source, length, and dosage (4 weeks to 24 weeks) were not significantly related to the magnitude of FBG reduction. However, anthocyanins from fruit extracts/powder also demonstrated a higher reduction in FBG. The Grade assessment indicated that our findings about HbA_1c_ and FBG were high quality and about 2-h postprandial glucose was moderate quality.

The mechanisms by which anthocyanins reduce glycemic responses are ascribed to the ability of anthocyanins to inhibit the key enzyme α-glucosidase, which is responsible for the conversion of sucrose to glucose during digestion in the intestinal epithelium (66, 67). Besides, anthocyanins also affect glucose absorption in the intestine, mediated by active Na-dependent and independent transport via sodium-glucose co-transporter 1 (SGLT1) and glucose transporter 2 (GLUT2), respectively, in Caco-2 cells of the intestine and HepG2 cells (20, 66, 67). Moreover, consumption of anthocyanins was found to promote glycogen synthesis and lower gluconeogenesis in HepG2 cells and adipocytes by upregulating peroxisome proliferator-activated receptor-γ (PPARγ), a hormone that regulates adiponectin and the transcription of proteins involved in glucose and fatty acid cellular uptake, leading to lower FBG (68–72). Yan et al. (68) reported a higher adiponectin level after intervention with mulberry anthocyanin extract, which was found in lower amounts in the serum of patients with T2DM and closely related to the sensitivity of insulin (50). Although in our study no significant effects of anthocyanins on insulin resistance were observed, few of the studies included in the meta-analysis found that patients with T2DM could benefit from the intake of anthocyanins by reporting decreased fasting insulin and HOMA-IR levels (51, 53, 56). The mechanisms elucidated indicate that chronic hyperglycemia causes insulin resistance, which inhibits insulin from decreasing serum glucose levels in humans. The intake of anthocyanins has also been described to activate the phosphorylation of protein kinase B (AKT), mitogen-activated protein (MAP) kinase pathway, phosphatidylinositol-e-kinase (PI3K), and AMP-activated protein kinase pathways, leading to improvement in insulin sensitivity requiring lower levels of insulin to lower blood glucose. However, the underlying mechanisms involved have not been elucidated (6, 18, 68). Therefore, more RCTs are needed to have a better understanding of the underlying mechanisms of the effects of anthocyanins on insulin resistance in patients with T2DM. Long-term hyperglycemia induces inflammation in patients with T2DM, characterized by the formation of glycosylation end products of non-enzymatic glucose reactions with proteins or lipoproteins in the arterial walls and low-density lipoprotein particles in the blood, leading to dyslipidemia, which is a notable risk factor of cardiovascular disease in patients with T2DM. Dyslipidemia is identified by the imbalance of lipids including elevated concentration of TG and apo B-containing lipoproteins, lower HDL, and higher LDL cholesterol (50). In our results, TG and LDL cholesterol were found to have significant reductions with the consumption of anthocyanins (Figures 3A,D), and the evidence was graded as moderate quality. There was no significant effect of anthocyanin intervention on total and HDL cholesterol, but the subgroup analysis revealed that the pure anthocyanin supplemented group reduced total cholesterol level and increased HDL more than the intervention with anthocyanins from the fruit extracts group. The difference could be associated with the presence of different polyphenols and other bioactive components in the fruit extracts that also induce varying effects on the blood lipid profile. Overall, anthocyanins were effective in improving lipid profile status in patients with T2DM based on our findings for TG and LDL. Yang et al. (24) also indicated substantial improvements in lipid profile with the introduction of anthocyanins (from purified supplements and fruit extracts), including a reduction of TG, TC, and LDL cholesterol as well as an increase in HDL cholesterol levels in both healthy and cardiometabolic diseased populations. Similarly, Neyestani et al. (28) reported a significant effect of anthocyanins on all lipid profiles in healthy adults and patients with different health conditions such as obesity, metabolic syndrome, hyperlipidemia, and T2DM. Tiwari et al. (27) demonstrated a significant reduction in TC, TG, and LDL, but not in HDL in preclinical studies involving subjects who were overweight/obese, had pre-diabetes, and T2DM (human and animal). The mechanism behind changes in different lipid markers by consumption of anthocyanins is related to improving the functionality of HDL particles and reversal in cholesterol transport (73). Anthocyanins were reported to improve the outflow capacity of HDL particles by inhibiting cholesteryl ester transfer protein (CETP) in dyslipidemia subjects (74). Furthermore, in another study, the antioxidant effect of anthocyanins improved paraoxonase-1 activity in HDL particles, which led to enhanced functionality of HDL particles in hypercholesteremic subjects (75). One study stated that the possible mechanism for improvement in lipid profile by anthocyanins is associated with its effect on lipid peroxidation (29). Moreover, Yao et al. (76) stated that anthocyanins including cyanidin-3-glucoside and peonidin-3-glucoside could intensify the luminal precipitation of cholesterol in human Caco-2 cells, suppressing cholesterol uptake. The evidence suggests the beneficial effects of anthocyanins on lipid profile in patients with T2DM. However, more studies are required to understand the varied effects of anthocyanin on the accumulation, deposition, and storage of different types of cholesterol and triglycerides in different types of cells and tissues.

Our findings indicated that anthocyanin consumption leads to a reduction in systolic and diastolic blood pressure, but the results were not significant, and between-study heterogeneity was observed among the included trials (Figures 4A,B). Consistently, another meta-analysis and systematic review also reported that the effects of anthocyanins on both healthy people and those with cardiometabolic diseases (24) on blood pressure (SBP and DBP) was not statistically significant and had heterogeneity of 87.90% and 88.20%, respectively. Other evidence from studies on the effect of anthocyanins on blood pressure was also inconsistent. For instance, the consumption of pomegranate juice, a rich source of anthocyanins led to blood pressure reductions (77). Another study revealed that 500 mg/day consumption of dietary anthocyanins by the general population led to an 8% reduction in the risk of hypertension (78). Similarly, two studies using berries as the major source of anthocyanins reported positive effects of anthocyanins in lowering systolic blood pressure and diastolic blood pressure (26, 79). However, in a meta-analysis of six studies based on RCTs, the consumption of blueberries did not lower blood pressure (systolic and diastolic) (80). Two studies conducted on the effect of anthocyanins on blood pressure (25, 81) concluded that anthocyanins had no substantial effect on the blood pressure of the subjects. The inconsistencies in these findings are owing to the varying dosage of anthocyanins used and the varied combination of anthocyanins and other bioactive compounds (including soluble and insoluble dietary fiber) present in the fruits and extracts utilized, which have varying impacts on the blood pressure levels (20). The median intervention dose of anthocyanins in the analyzed RCTs in our study is 320 mg per day, this effective dosage finding has been corroborated by Sandoval et al. (20), who reported the intake of 200–400 mg of anthocyanins per day to considerably reduce FBG levels. However, for increased confidence, more studies need to be analyzed to understand the dosage effect of anthocyanins in T2DM for different fruit extracts and effective dosage. In addition, the activity of anthocyanins is subject to environmental conditions, including pH and temperature, that affect the bioavailability of anthocyanins. Anthocyanins are not stable at neutral pH and physiological temperature and can interact with other food components, such as proteins and carbohydrates, affecting their bioavailability on consumption (2, 82). This means that they are susceptible to gastrointestinal pH (highly acidic in gastric and alkaline in intestinal phases), enzymes, and gut microbes and these factors lead to the absorption and release of limited amounts of anthocyanins in blood and urine (2, 83). For instance, the bioavailability of cyanidin 3-O-glucoside is only 12% in humans (84). All these reasons limit the application of anthocyanins in the treatment of T2DM. In addition, the beneficial effects of anthocyanins tend to be underestimated as most of the anthocyanins consumed do not get absorbed in the stomach or small intestine and reach the colon in their intact form (2). The small amount of absorbed anthocyanins is metabolized further in the liver or kidneys (2, 85). These metabolites of anthocyanins, including gallic acid, protocatechuic acid, benzoic acids, vanillic acid, syringic acid, and phase 2 metabolites, are highly bioavailable (2, 84, 86–88). For instance, the primary gut metabolite of cyanidin 3-glucoside is protocatechuic acid, which inhibits the generation of nitric oxide and the release of TNF-α, resulting in anti-inflammatory effects (89). Another example is the main gut metabolite of delphinidin-3-glucoside-gallic acid. It plays an important role in inhibiting both inflammation and atherosclerosis by reducing the secretion of monocyte chemoattractant protein-1, intercellular adhesion molecule 1, and vascular cell adhesion molecule 1 by endothelial cells (89). In addition, the anthocyanin metabolites gallic acid, 3-o-methylgallic acid, and 2,4,6-trihydroxybenzaldehyde have been proven to substantially reduce human colon cancer cell viability (90). All of these metabolites exhibit beneficial health effects, however, the rate and extent of the absorption and metabolism of the anthocyanins and production of metabolites in the gut, is not well understood. Therefore, it is better to develop an effective delivery system to transport anthocyanins directly to the target tissue or organ, slow their release, and maximize their beneficial effects.

## 5. Conclusion

The meta-analysis of 13 RCTs illustrated that anthocyanins were beneficial in improving glycemic parameters in patients with T2DM, including HbA_1c_, FBG, and 2-h postprandial glucose values. Our GRADE assessment also rated the evidence for the effect of anthocyanins in reducing HbA_1c_ and FBG with high certainty and 2-h postprandial glucose level with moderate certainty. Furthermore, anthocyanins from fruit extracts or powder showed a significantly higher effect in lowering HbA_1c_ than pure supplements. In terms of insulin resistance, no significant results were observed regarding the effects of anthocyanins on both fasting insulin and HOMA-IR. More long-term RCTs studying varied dosages of anthocyanins are required to further evaluate these effects and underlying mechanisms. Anthocyanins were effective in improving the lipid profile of patients with T2DM including reducing the levels of TG and LDL cholesterol. This further supported anthocyanins as an effective alternative for the prevention and treatment of disorders of lipid metabolism in patients with T2DM. Thus, the consumption of anthocyanins has the potential to delay the progress of T2DM and improve the lipid profile related to cardiovascular diseases. However, further studies are needed to explain the varied effects of anthocyanin consumption on the individual lipid components, especially TC and HDL cholesterol, as we did not observe significant results in either of them. The underlying mechanisms involved in the activity of anthocyanins in T2DM and cardiovascular disease patients are vaguely understood, but more *in-vitro*, *in-vivo*, and animal studies are also needed to understand the role of different anthocyanins in these health conditions. Overall, we concluded that increased anthocyanin consumption, especially anthocyanin-rich fruit extracts or powder, is a promising remedy for managing glycemic markers and improving lipid profile in patients with T2DM. Increasing the number of human clinical trials and meta-analyses in this area will enable us to elucidate the optimal dosage, as well as the intervention duration required for pure anthocyanin supplements in patients with T2DM. In addition, the utilization of fruit powders and extracts could provide sustainable low-cost alternatives for mitigating the incidence and reducing the rate of progression of T2DM and related cardiovascular diseases. Further studies are required to map out the individual, complementary, and synergistic effects of different bioactive molecules including anthocyanins, flavonoids, and dietary fiber present in fruit powders and extracts, which will enable the designing of effective supplements and dietary intervention dosages and formulations. Moreover, it is necessary to investigate nanocarriers or other effective delivery systems to transport anthocyanins to the target tissue or organ in order to increase the stability, bioavailability, and therapeutic effects of anthocyanins in patients with T2DM.

## Data availability statement

The original contributions presented in the study are included in the article/Supplementary material, further inquiries can be directed to the corresponding author.

## Author contributions

TM: conceptualization, investigation, methodology, formal analysis, writing—original draft, and writing—review and editing. FA: investigation, methodology, writing—original draft, and writing—review and editing. MM: conceptualization, supervision, methodology, project administration, and writing—review and editing. All authors contributed to the article and approved the submitted version.
