# Supplementary material for: Effects of anthocyanin supplementation in diet on glycemic and related cardiovascular biomarkers in patients with type 2 diabetes: a systematic review and meta-analysis of randomized controlled trials
Source: Front Nutr. 2023 Sep 22;10:1199815. doi: 10.3389/fnut.2023.1199815 (PMC10556752; doi:10.3389/fnut.2023.1199815)
Supplement: Supplementary file 1 [file Data_Sheet_1.docx]

Supplementary Material


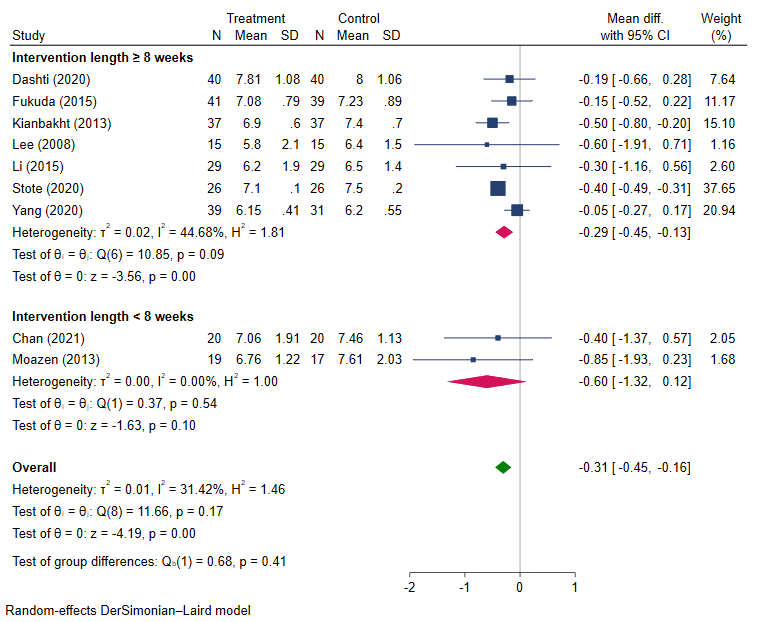


**Supplementary Figure 1A.** Subgroup analysis of the effect of anthocyanins on HbA1c on intervention length ≥ 8 weeks and intervention difference < 8 weeks.

Diamond represented the pooled effect estimate for overall analysis. Data were represented as mean difference with 95% CI, using the random-effects DerSimonian-Laird model. Interstudy heterogeneity quantified by *I*^2^ with significant p < 0.05. Test of homogeneity (θ_i_ = θ_j_) of study-specific effects sizes, with the chi-squared test statistic rejected if p < 0.05.


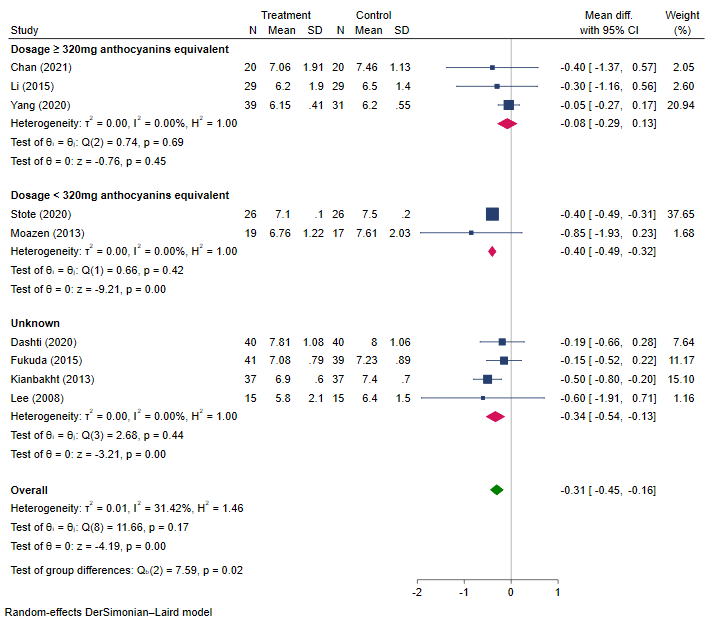


**Supplementary Figure 1B.** Subgroup analysis of the effect of anthocyanins on HbA1c on dosage difference (≥ 320 mg anthocyanins equivalent, < 320 mg anthocyanins equivalent, and unknown anthocyanins amount equivalent).

Diamond represented the pooled effect estimate for overall analysis. Data were represented as mean difference with 95% CI, using the random-effects DerSimonian-Laird model. Interstudy heterogeneity quantified by *I*^2^ with significant p < 0.05. Test of homogeneity (θ_i_ = θ_j_) of study-specific effects sizes, with the chi-squared test statistic rejected if p < 0.05.


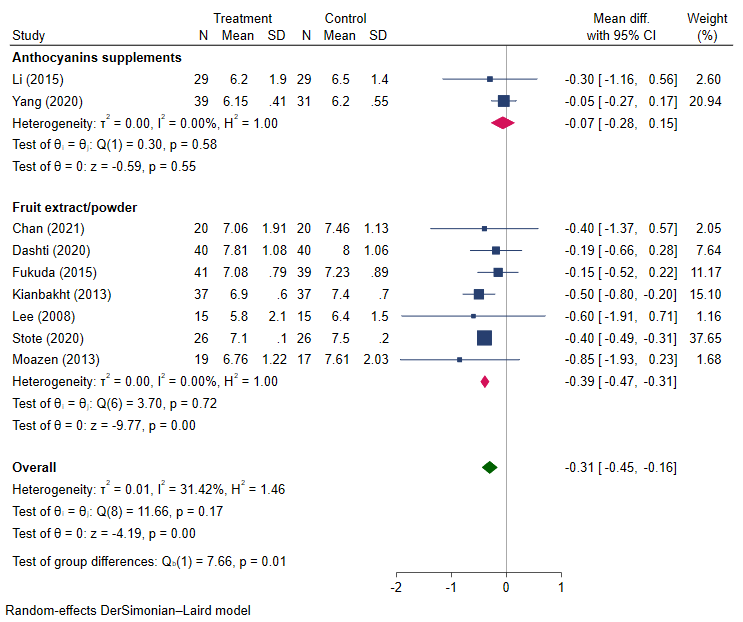


**Supplementary Figure 1C.** Subgroup analysis of the effect of anthocyanins on HbA_1c_ on anthocyanins supplements and fruit extracts/powder.

Diamond represented the pooled effect estimate for overall analysis. Data were represented as mean difference with 95% CI, using the random-effects DerSimonian-Laird model. Interstudy heterogeneity quantified by *I*^2^ with significant p < 0.05. Test of homogeneity (θ_i_ = θ_j_) of study-specific effects sizes, with the chi-squared test statistic rejected if p < 0.05.


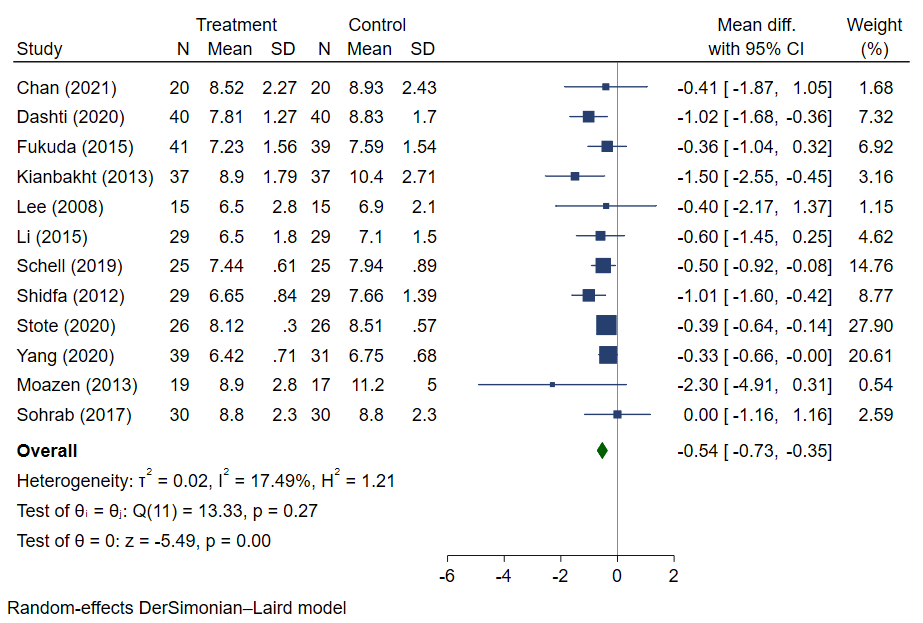


**Supplementary Figure 2A.** The effect of anthocyanins on fasting blood glucose after removing the study done by Soltani et al. (52)

Diamond represented the pooled effect estimate for overall analysis. Data were represented as mean difference with 95% CI, using the random-effects DerSimonian-Laird model. Interstudy heterogeneity quantified by *I*^2^ with significant p < 0.05. Test of homogeneity (θ_i_ = θ_j_) of study-specific effects sizes, with the chi-squared test statistic rejected if p < 0.05.


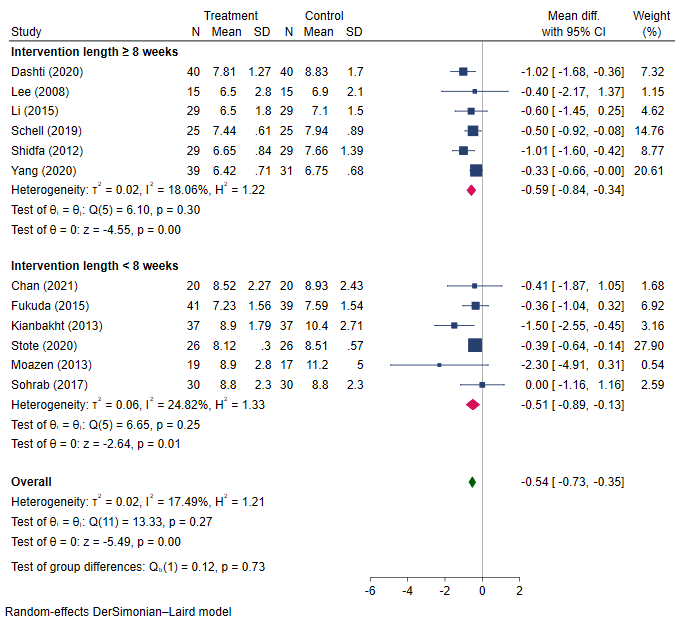


**Supplementary Figure 2B.** Subgroup analysis of the effect of anthocyanins on fasting blood glucose on intervention length ≥ 8 weeks and intervention difference < 8 weeks.

Diamond represented the pooled effect estimate for overall analysis. Data were represented as mean difference with 95% CI, using the random-effects DerSimonian-Laird model. Interstudy heterogeneity quantified by *I*^2^ with significant p < 0.05. Test of homogeneity (θ_i_ = θ_j_) of study-specific effects sizes, with the chi-squared test statistic rejected if p < 0.05.


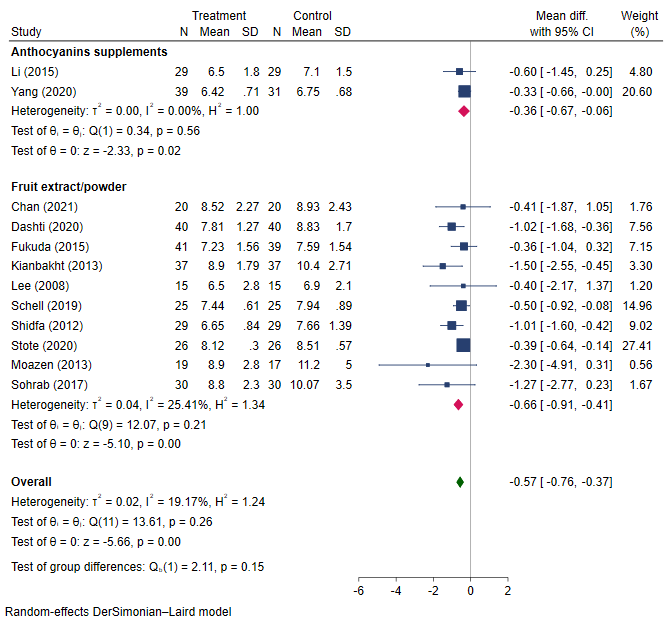


**Supplementary Figure 2C.** Subgroup analysis of the effect of anthocyanins on fasting blood glucose on anthocyanins supplements and fruit extracts/powder.

Diamond represented the pooled effect estimate for overall analysis. Data were represented as mean difference with 95% CI, using the random-effects DerSimonian-Laird model. Interstudy heterogeneity quantified by *I*^2^ with significant p < 0.05. Test of homogeneity (θ_i_ = θ_j_) of study-specific effects sizes, with the chi-squared test statistic rejected if p < 0.05.


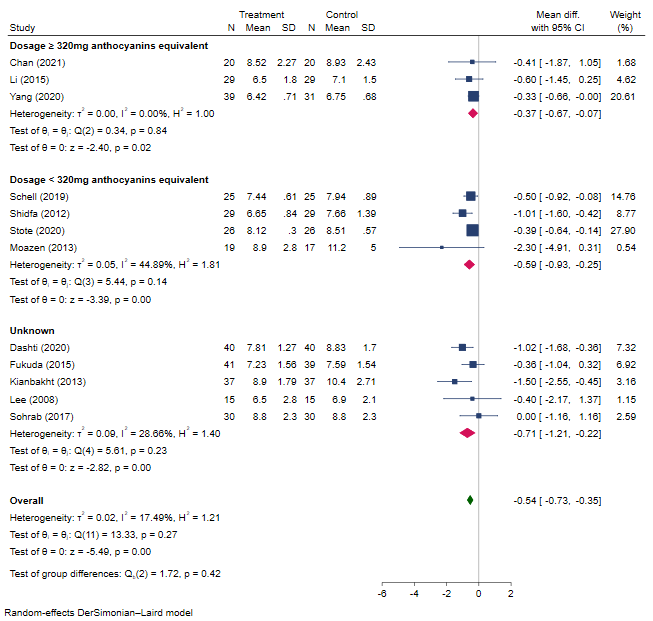


**Supplementary Figure 2D.** Subgroup analysis of the effect of anthocyanins on fasting blood glucose on dosage difference (≥ 320mg anthocyanins equivalent, < 320mg anthocyanins equivalent, and unknown anthocyanins amount equivalent).

Diamond represented the pooled effect estimate for overall analysis. Data were represented as mean difference with 95% CI, using the random-effects DerSimonian-Laird model. Interstudy heterogeneity quantified by *I*^2^ with significant p < 0.05. Test of homogeneity (θ_i_ = θ_j_) of study-specific effects sizes, with the chi-squared test statistic rejected if p < 0.05.


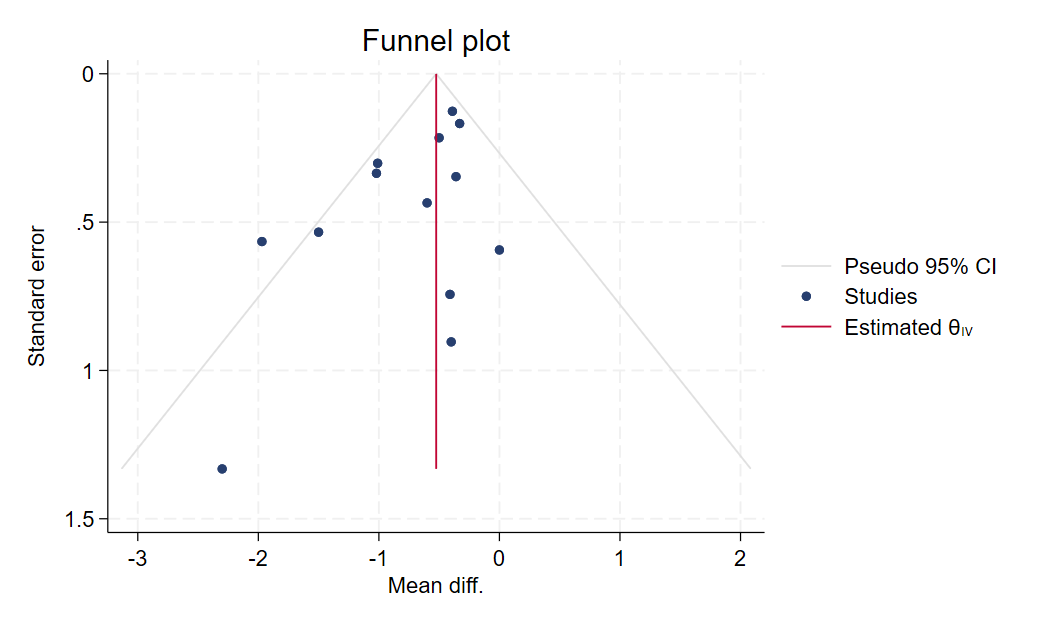


**Supplementary Figure 2E.** Funnel plot of the effect of anthocyanins on fasting blood glucose


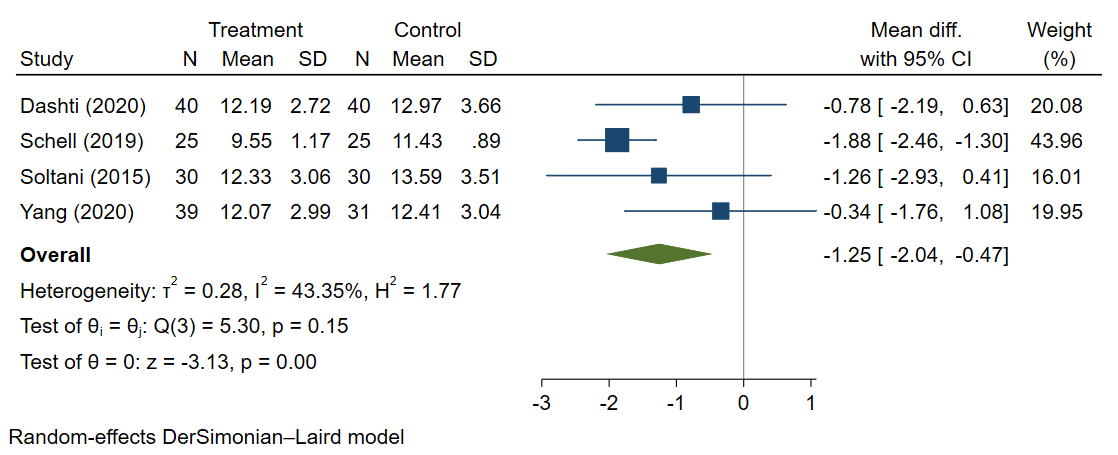


**Supplementary Figure 3.** The effect of anthocyanins on 2-hour postprandial glucose after removing the study done by Kianbakht et al. (49)

Diamond represented the pooled effect estimate for overall analysis. Data were represented as mean difference with 95% CI, using the random-effects DerSimonian-Laird model. Interstudy heterogeneity quantified by *I*^2^ with significant p < 0.05. Test of homogeneity (θ_i_ = θ_j_) of study-specific effects sizes, with the chi-squared test statistic rejected if p < 0.05.


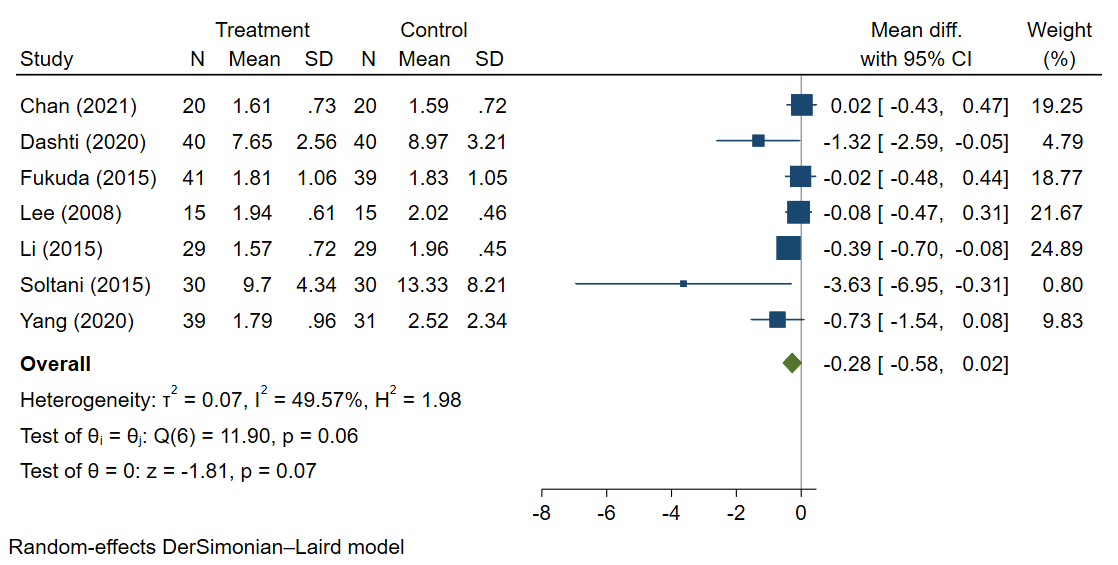


**Supplementary Figure 4.** The effect of anthocyanins on triglycerides after removing the study done by Stote et al. (53)

Diamond represented the pooled effect estimate for overall analysis. Data were represented as mean difference with 95% CI, using the random-effects DerSimonian-Laird model. Interstudy heterogeneity quantified by *I*^2^ with significant p < 0.05. Test of homogeneity (θ_i_ = θ_j_) of study-specific effects sizes, with the chi-squared test statistic rejected if p < 0.05.


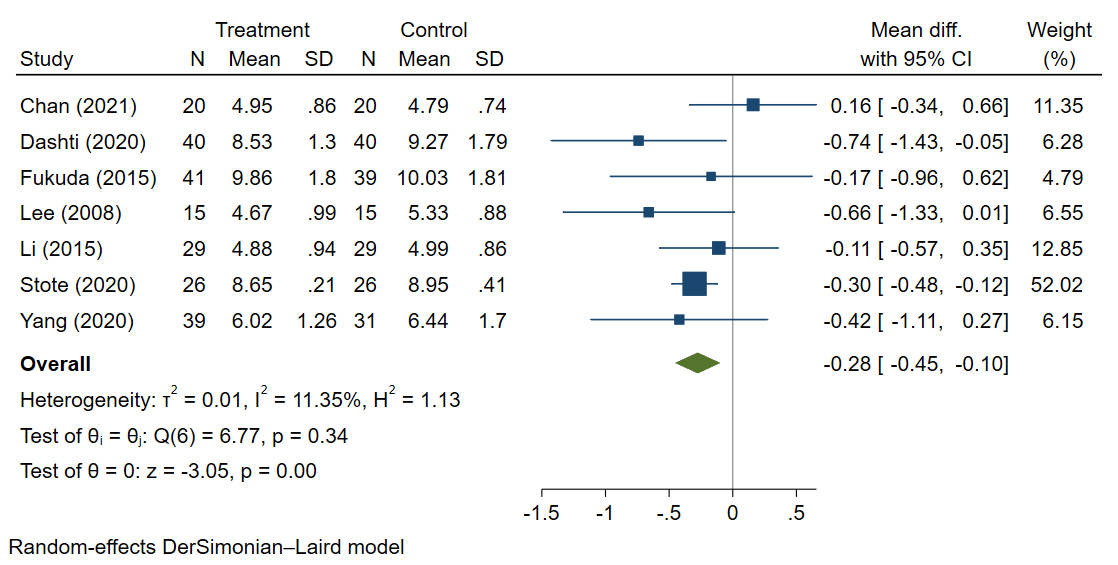


**Supplementary Figure 5A.** The effect of anthocyanins on total cholesterol after removing the study done by Schell et al. (51)

Diamond represented the pooled effect estimate for overall analysis. Data were represented as mean difference with 95% CI, using the random-effects DerSimonian-Laird model. Interstudy heterogeneity quantified by *I*^2^ with significant p < 0.05. Test of homogeneity (θ_i_ = θ_j_) of study-specific effects sizes, with the chi-squared test statistic rejected if p < 0.05.

**Supplementary Figure 5B.** Subgroup analysis of the effect of anthocyanins on total cholesterol on anthocyanins supplements and fruit extracts.

Diamond represented the pooled effect estimate for overall analysis. Data were represented as mean difference with 95% CI, using the random-effects DerSimonian-Laird model. Interstudy heterogeneity quantified by *I*^2^ with significant p < 0.05. Test of homogeneity (θ_i_ = θ_j_) of study-specific effects sizes, with the chi-squared test statistic rejected if p < 0.05.

**Supplementary Figure 6.** The effect of anthocyanins on HDL cholesterol after removing studies done by Dashti et al. (9), Lee et al. (56), and Li et al. (50).

Diamond represented the pooled effect estimate for overall analysis. Data were represented as mean difference with 95% CI, using the random-effects DerSimonian-Laird model. Interstudy heterogeneity quantified by *I*^2^ with significant p < 0.05. Test of homogeneity (θ_i_ = θ_j_) of study-specific effects sizes, with the chi-squared test statistic rejected if p < 0.05.


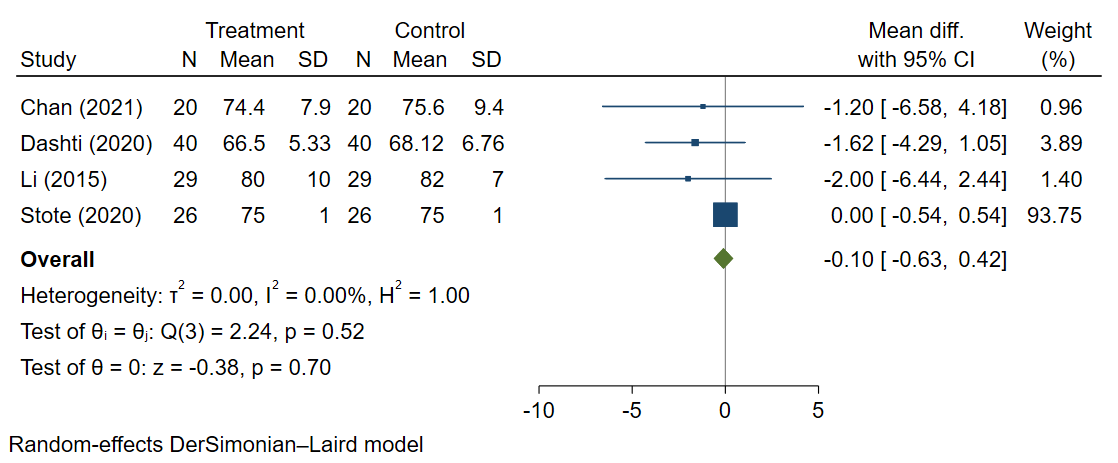


**Supplementary Figure 7.** The effect of anthocyanins on diastolic blood pressure after removing the studies done by Lee et al. and Schell et al.

Diamond represented the pooled effect estimate for overall analysis. Data were represented as mean difference with 95% CI, using the random-effects DerSimonian-Laird model. Interstudy heterogeneity quantified by *I*^2^ with significant p < 0.05. Test of homogeneity (θ_i_ = θ_j_) of study-specific effects sizes, with the chi-squared test statistic rejected if p < 0.05.
